# Brazilian Guidelines on evaluation and clinical management of Nephrolithiasis: Brazilian Society of Nephrology

**DOI:** 10.1590/2175-8239-JBN-2024-0189en

**Published:** 2025-03-10

**Authors:** Mauricio de Carvalho, Ana Cristina Carvalho de Matos, Daniel Rinaldi dos Santos, Daniela Veit Barreto, Fellype Carvalho Barreto, Fernanda Guedes Rodrigues, Igor Gouveia Pietrobom, Lucas Gobetti da Luz, Natasha Silva Constancio, Samirah Abreu Gomes, Ita Pfeferman Heilberg

**Affiliations:** 1 Universidade Federal do Paraná, Hospital de Clínicas, Curitiba, PR, Brazil.; 2 Pontifícia Universidade Católica do Paraná, Disciplina de Nefrologia, Curitiba, PR, Brazil.; 3 Universidade Federal de São Paulo, Disciplina de Nefrologia, Escola Paulista de Medicina, São Paulo, SP, Brazil.; 4 Faculdade de Medicina do ABC, Santo André, SP, Brazil.; 5 Universidade Federal de São Paulo, Pós-Graduação em Nutrição, São Paulo, SP, Brazil.; 6 Hospital Moinhos de Vento, Departamento de Nefrologia, Porto Alegre, RS, Brazil.; 7 Hospital Unimed Vale do Sinos, Novo Hamburgo, RS, Brazil.; 8 Hospital Regional Alto Vale e Associação Renal Vida, Rio do Sul, SC, Brazil.; 9 Universidade de São Paulo, Faculdade de Medicina, São Paulo, SP, Brazil.

**Keywords:** Nephrolithiasis, Kidney Calculi, Urolithiasis

## Abstract

The prevalence of nephrolithiasis has been increasing in recent years, affecting appro­ximately 10% and 15% of the population. Kidney stone disease is associated with syste­mic comorbidities such as cardiovascular dis­ease, diabetes mellitus, and obesity. The first Nephrolithiasis Guideline by the Brazilian Society of Nephrology was published in 2002, and since then, the accumulation of new clinical studies and guidelines has justified a review of the subject. This updated document, prepared by the Nephrolithiasis Committee of the Brazilian Society of Nephrology, reflects the advances in the management of patients with kidney stones. The guideline aims to provide recommendations for the diagnosis, prevention, and treatment of nephrolithiasis, based on the best available evidence. Topics covered include clinical evaluation, laboratory and imaging tests, as well as dietary and pharmacological interventions, and follow-up strategies.

## Introduction

Nephrolithiasis is one of the most common clinical and surgical conditions, characterized by the appearance of solid crystalline structures within the urinary system. The prevalence of kidney stone disease continues to increase globally, especially in recent years, across both sexes and all ethnic groups. It is estimated to affect 10% to 15% of the population, with a higher frequency in men, at a ratio of 2–3:1 compared to women. The peak incidence occurs between the third and fifth decades of life, with recurrence rates, if untreated, reaching up to 50% within 10 years^1^. Data analysis from the United States National Health and Nutrition Examination Survey (NHANES), conducted between 2015 and 2018 and involving more than 10,000 patients over the age of 20, revealed a prevalence of 11% (95% CI: 10.1–12). The 12-month incidence was 2.1% (95% CI: 1.5–2.7)^2^.

In addition, recent evidence shows that nephrolithiasis is related to other systemic conditions, mainly cardiovascular disease, diabetes mellitus, and obesity. Nephrolithiasis may also be associated with the development of chronic kidney disease, particularly in females and overweight patients^3^.

The progressive increase in prevalence and the costs associated with nephrolithiasis have rendered it one of the most common and impactful conditions currently seen in medical practice. Prevention remains the most effective way to avoid urinary stones, especially given their high recurrence rate.

The evaluation of kidney stone patients requires a comprehensive clinical approach aimed at identifying environmental, metabolic, and/or genetic factors related to stone formation. Furthermore, imaging studies are needed to assess and monitor the presence of stones in the urinary tract, as well as laboratory analyses (biochemical composition of blood and urine, stone composition analysis) to target therapeutic strategies, both nutritional and pharmacological.

It is unfortunate that none of the most influential nephrology societies (ASN, ISN, ERA) have issued specific guidelines on urolithiasis to date^4^. In 2002, the first Nephrolithiasis Guideline by the Brazilian Society of Nephrology was published^5^. Over this extended period, updates to various guidelines on the subject, along with the publication of numerous clinical studies, have prompted the need to review the topic. This work was conducted by the Nephrolithiasis Committee of the Brazilian Society of Nephrology and aims to reflect advances in the management of patients with kidney stones. It should be emphasized that this document reflects the best selected and available evidence for the members of this committee. However, guidelines do not replace clinical expertise in decision-making for individual patients, but rather help to guide these decisions.

Although not explicitly suggested, the publication of this guideline constitutes a call to action for the nephrologist’s co-management in the longitudinal and holistic care of patients with urinary stone disease. And, ideally, in cooperation with other healthcare professionals, such as primary care physicians, internists, nutritionists, urologists, among others^4^.

## Methods

The information presented in this document was obtained through literature review, primarily in English. For this Guideline, evidence was identified, compiled, and analyzed by a structured evaluation of the articles.

The recommendations were based, as far as possible, on the most recent literature since the last guideline was published in 2002^5^. The search range was extended until June 2024, including some of the following terms in the title or abstract: “nephrolithiasis”, “urolithiasis”, “kidney stone”, “renal stone”, “urinary stone”, “urinary calculi”.

The search was primarily directed, though not exclusively, towards studies that represented high levels of evidence, such as systematic reviews with meta-analysis, randomized clinical trials, and non-randomized prospective comparative studies. In addition, references from recently published guidelines were also reviewed. Separate literature reviews were conducted for each of the main topics^6^.

The 2011 Oxford Center for Evidence-Based Medicine Levels of Evidence scoring system was used to assess the level of evidence for the recommendations included in the document^7^, as shown in Table 1. The recommendations were based on a specialized review of the literature and represent the consensus of this guideline’s authors. All participants involved submitted statements of potential conflicts of interest.

**Table 1:** Classification system – levels of evidence

Degree of recommendation	Description
A	High-quality randomized controlled trials
B	Well-conducted observational studies and low-quality randomized clinical trials
C	Case-control studies and case series
D	Expert opinion
Level of Evidence	Description
1	Evidence derived from meta-analyses of randomized clinical trials or at least one well-conducted randomized clinical trial
2	Evidence derived from at least one well-conducted clinical trial or high-quality observational studies with consistency among them
3	Evidence derived from observational studies, case series, or case reports
4	Evidence derived from expert opinions based on clinical experience, descriptive studies, or expert committee reports

## Acute Renal Colic

### Clinical Picture

Nephrolithiasis may manifest as completely asymptomatic, being incidentally diagnosed through imaging tests, or it may only cause a vague flank pain. However, the classic presentation is acute ureteric colic, which usually begins with sudden, intense, and unilateral pain. This pain waxes and wanes in intensity, developing in waves or paroxysms that typically last from 20 to 60 minutes^8^. The severity of the pain is more closely related to the speed at which the obstruction develops than to its intensity. This pain is commonly located in the lumbar region, the flank, or the iliac fossa. It is not relieved by rest or changes in position and may radiate to the ureteral tract, bladder region, and external genitalia. Other symptoms may include dysuria and gross hematuria. Gastrointestinal symptoms could also occur to varying degrees, such as nausea (44%), vomiting (51%), anorexia (54%), constipation, or diarrhea (10%)^9^.

Other clinical presentations of nephrolithiasis include micro or macroscopic, non-glomerular (isomorphic) hematuria. However, hematuria may not be detected in approximately 10% to 30% of patients with renal colic^10^. The interval between the onset of acute pain and the moment of urinalysis is a factor that could impair the sensitivity of hematuria. In a retrospective study of more than 450 patients with acute ureterolithiasis documented by computed tomography (CT), hematuria was present in 95% on day 1, and 65% to 68% on days 3 and 4^11^.

Nephrolithiasis may also present as spontaneous passage of stones without the presence of pain, or through recurrent urinary tract infections, especially those caused by bacteria of the genus *Proteus*. The combination of low back pain, fever, chills, and sepsis may occur in calculous obstructive pyelonephritis, a condition with high morbidity and mortality.

On physical examination, tachycardia, pallor, sweating, tenderness on palpation in the costovertebral angle region (Giordano’s sign, with a sensitivity of 15%, specificity of 99%, and likelihood ratio of +30), and mild abdominal distension may be observed, but with no signs of peritoneal irritation^12^. The clinical picture is suggestive, but it is important to carry out a differential diagnosis with gastrointestinal, gynecological, and urological pathologies, vascular conditions, and other medical causes of acute abdomen.

The STONE score is a clinical prediction tool that helps identify patients with acute abdominal pain who have a very low or very high probability of uncomplicated ureteral stone. Thus, it helps in the decision-making process of complementing the investigation for alternative diagnoses and the request for imaging tests. The variables analyzed in the protocol are: sex, race, duration of pain, nausea and/or vomiting, and presence of hematuria^13^.

### Summary and Recommendations

The association of recent onset abdominal pain (up to 12 hours), tenderness in the lower back or ureteral region, and the presence of hematuria is suggestive of acute renal colic. Therefore, obtaining a detailed clinical history and a thorough physical examination are of utmost importance (Grade of recommendation: B; Level of evidence: 2).

Diagnostic scores, such as the STONE score, may be used as an aid in decision-making when there is uncertainty in the diagnosis and the need for immediate treatment (Grade of recommendation: B; Level of evidence: 2).

### Additional Investigation

Confirmation of the presence of urinary stones and their location is recommended. Immediate imaging evaluation is indicated for patients with fever, those with a solitary kidney, and when the diagnosis is uncertain. However, pain relief or any other emergency measure should not be delayed for imaging tests to be performed^14^.

Plain abdominal radiography (X-ray) is frequently used because approximately 90% of kidney stones have a radiopaque density. To be visible, the stone must be at least 2 mm in its largest diameter. However, plain X-rays have limited sensitivity for detecting ureteral stones. Some studies report sensitivity lower than 50%. Despite this, they are still considered useful tools for monitoring the progression of radiopaque stones, especially when interpreted in conjunction with ultrasonography^15,16^.

Ultrasound can detect all types of kidney stones, regardless of radiopacity, in addition to evaluating the presence and degree of potential hydronephrosis. This modality of examination can be performed both during episodes of acute renal colic and during pregnancy. Ultrasound has reduced sensitivity for detecting ureteral stones (45% sensitivity and 94% specificity) and is subject to dependence on the equipment used and the operator’s ability to interpret the images^17^.

Computed tomography (CT) is the gold standard for diagnosing urinary stone disease. A meta-analysis revealed that low-dose CT screening diagnosed urolithiasis with a sensitivity of 93.1% (95% CI: 91.5–94.4) and specificity of 96.6% (95% CI: 95.1–97.7%). Additionally, it allows the abdomen to be evaluated in a matter of minutes without the need for contrast, it can diagnose diseases unrelated to the urinary tract, and visualize almost all types of stones (whether radiopaque or not)^18^. It also enables the use of density, measured in Hounsfield units, to estimate the stone composition (on an increasing density scale - uric acid, struvite, cystine, calcium oxalate monohydrate, and hydroxyapatite) and its response to treatment (stones with a density greater than 1,000 Hounsfield units are difficult to fragment by extracorporeal lithotripsy)^19^. Its disadvantages include greater radiation exposure and higher costs^20^. Magnetic resonance imaging (MRI) has limited application in the investigation of urinary calculi. Its use is reserved only for special situations in pregnant women and children^21^. The complete blood count usually remains unchanged or may show mild leukocytosis during the acute phase, with no significant left shift. However, in elderly patients with a marked increase in C-reactive protein (CRP) levels, associated infection should be considered, and antibiotic treatment may be necessary^22^. The serum creatinine concentration is usually normal, except in situations such as obstructive calculi in a solitary kidney, bilateral ureteral obstruction, the presence of large bladder stones, or impacted urethral stones. Leukocytes may be found in the urinalysis; however, the presence of positive nitrite and bacteria in the sediment examination should raise the suspicion of an associated infection. Microscopic or macroscopic hematuria is common in renal colic, but its absence does not exclude the diagnosis.

**Figure 1:**
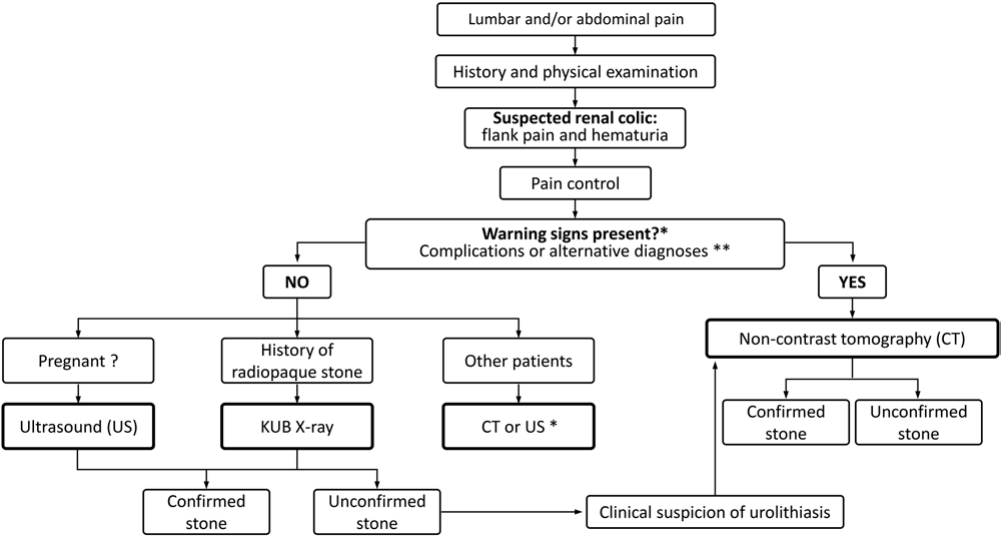
Investigation of suspected renal colic.

Figure 1 summarizes the steps for investigating suspected renal colic.

Notes – *Alternative diagnoses with clinical relevance: Aortic aneurysm rupture or dissection, malignancy (renal, ovarian), diverticulitis, appendicitis, cholecystitis, intestinal obstruction, ovarian/testicular torsion, ectopic pregnancy, renal thrombosis/infarction, retroperitoneal hematoma, pulmonary embolism, pneumonia. **Warning signs: First episode >50 years old, fever, hemodynamic changes, single kidney, renal dysfunction, atypical renal colic presentation.

### Summary and Recommendations

Immediate imaging tests are indicated when there is uncertainty in the differential diagnosis with other causes of lower back pain or in the absence of hematuria, in cases of fever, or when the patient reports the presence of a solitary kidney. Ultrasound, in conjunction with plain abdominal radiography, can be considered the initial modality for renal colic (Grade of recommendation: A; Level of evidence: 1).

Computed tomography, preferably in a low-dose protocol, provides valuable information for diagnostic confirmation and therapeutic decision-making, with advantages in cases of identifying stones in the ureteral region, as well as determining the repercussion of the stone, suggesting obstruction or not. It should be noted that ultrasound can also indicate the presence of dilation due to stone obstruction (Grade of recommendation: A; Level of evidence: 1).

In emergency care, a brief biochemical evaluation is recommended, with creatinine measurement, complete blood count, and urinalysis. Patient age (>54 years old) and elevated CRP levels (>5 times the reference value) help to decide whether to prescribe antibiotics, in the case of suspected infection associated with acute renal colic (Grade of recommendation: C; Level of evidence: 4).

### Treatment

The pain of renal colic is believed to be caused by spasms of the smooth muscles of the ureter, associated with edema and inflammation resulting from the obstruction caused by the stone. In addition, increased peristalsis and pressure proximal to the stone contribute to the intensification of pain^23^. The two main classes of medication used for analgesia in renal colic are nonsteroidal anti-inflammatory drugs (NSAIDs) and opioids. By inhibiting prostaglandin synthesis, NSAIDs reduce inflammation and ureteral muscle hyperactivity. In a study published in 2016, 1500 adult patients were randomized to receive intramuscular diclofenac, intravenous morphine, or intravenous paracetamol. After 30 minutes, NSAIDs were 50% more effective in reducing pain compared to morphine, with no adverse events. A limitation of the study was the morphine dose (0.1 mg/kg/IV), which was lower than that typically used^24^. A recent meta-analysis showed that patients treated with NSAIDs achieved a reduction in pain scores similar to those treated with opioids, but with fewer side effects^25^. Ketoprofen is one of the commonly used NSAIDs, with good analgesic action and may be administered intravenously. Other NSAIDs, such as diclofenac, ibuprofen, or indomethacin, also have good levels of evidence supporting their use^26^.

However, it should be remembered that NSAIDs are absolutely or relatively contraindicated in situations such as kidney failure, severe peptic ulcer disease, and pregnancy – in these cases, opioids should be considered. Morphine is the classic representative of this class of drugs, and although it does not act on the pathophysiology of ureteral colic, it provides rapid, potent and titratable analgesic action. However, it has side effects such as nausea, constipation, among others, as well as urinary retention, respiratory depression, and, at higher doses^27^, hypotension. Tramadol hydrochloride causes less sedation, but with lower analgesic effect. Meperidine hydrochloride induces frequent vomiting, and its action may be prolonged or potentiated in kidney failure^28^. Antispasmodics, such as hyoscine, have a controversial and limited effect, even when used in combination with other analgesics^29^.

Although the United States and England have banned the use of dipyrone due to the rare occurrence of agranulocytosis, this agent is the most popular non-opioid drug used for analgesia in other countries, including Brazil. It is used either in combination with hyoscine or alone, with good results, despite side effects related to drowsiness.

Although rehydration is useful in hypovolemic patients with significant nausea and vomiting, or in those with suspected pre-renal acute kidney injury, intravenous hydration solely for the purpose of promoting forced passage of stones is not supported by the literature and should be avoided^30^.

Spontaneous passage of ureteral stones occurs in up to 80% of stones smaller than 5 mm. For stones larger than 7 mm, the chance is much lower, around 25% for those located in the proximal ureter, 45% for those in the middle ureter, and 70% for distal ureter calculi^31^.

Alpha-1-adrenergic receptor blockers (e.g. tamsulosin) have been used to facilitate the spontaneous passage of ureteral stones, especially those located in the distal ureter and smaller than 1 cm. Several studies on so-called medical expulsive therapy (MET), most of them randomized and controlled, but with a small number of patients, have shown that alpha-blockers accelerate stone passage, reduce pain, and consequently decrease the need for analgesics, with minimal side effects (hypotension, especially in the first dose)^32^. However, a multicenter study evaluating over 1,000 patients revealed that MET was similar to placebo in the number of interventions required to eliminate ureteral stones. In this study, 75% of the stones were <5mm in diameter, and the majority (65%) were located in the lower ureter^33^. More recently, a new meta-analysis of only randomized controlled trials showed a 44% greater chance of eliminating distal ureteral calculi of 5–10 mm with the use of tamsulosin^34^. Tamsulosin, as well as potassium citrate (at an average dose of 55 mEq/day), is also used in some protocols as complementary therapy after extracorporeal shock wave lithotripsy to accelerate the elimination of stone fragments^35^.

**Figure 2:**
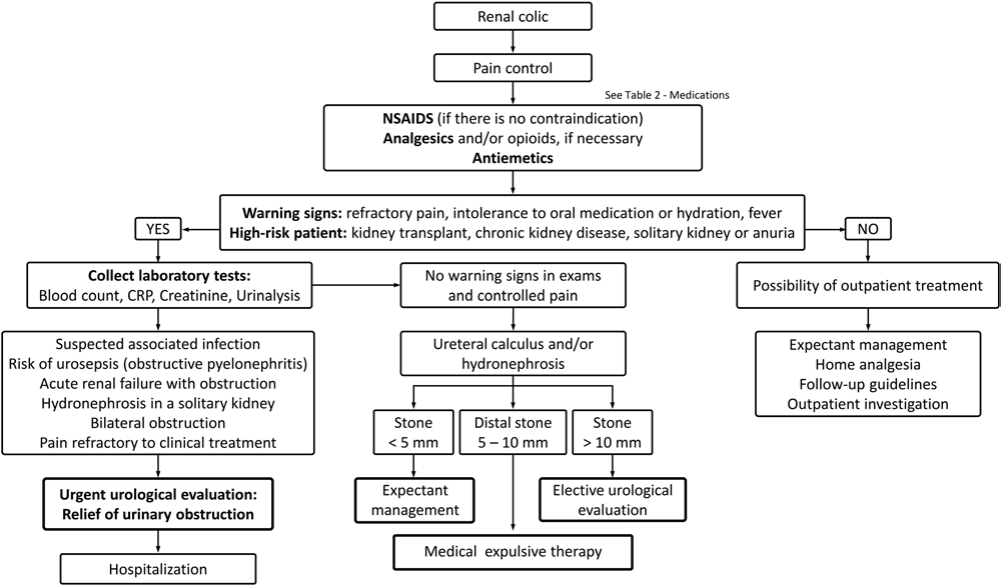
Renal colic management.

Figure 2 illustrates the steps in the management of renal colic, and Table 2 summarizes the main medications used in renal colic.

**Table 2:** Main medications used to treat renal colic in the emergency room

Class	Presentation (adult dose)	Observations (side effects)	Contraindications
***Analgesics***			
Paracetamol	**Tab. 500–750 mg:** 1 tab. PO 4-6x/day (Max dose: 4000 mg/day)	Hepatotoxicity, liver enzyme alterations	*Absolute*: hypersensitivity, *Relative*: G6PD deficiency, active liver disease
Dipyrone (Metamizole)	**Tab. 500–1000 mg:** (2 tabs. PO 4x/day) **Ampoule 500 mg/mL (2 mL):** 1–2 ampoules IV 4x/day	Anaphylaxis, hypotension, agranulocytosis	*Absolute*: hypersensitivity, *Relative*: pregnant and lactating women, bone marrow disease, G6PD deficiency
***Antispasmodics***			
Scopolamine	Tablet 10 mg: 1 to 2 Tablets 4x/day (Max dose PO: 60 mg/day) Ampoule 30 mg/mL (1 mL): 1 to 2 ampoules IV, IM or SC 4-6x/day (Max dose IV: 100 mg/day)	Xerostomia, blurred vision, urinary retention, drowsiness, fatigue, psychiatric disturbances (agitation, confusion, hallucinations). Complications often occur in the elderly and with higher doses	*Absolute*: hypersensitivity, glaucoma, megacolon, paralytic ileus, benign prostatic hyperplasia with urinary retention, gastrointestinal stenotic lesions, myasthenia gravis, tachycardia due to heart failure
***Non-Steroidal Anti-Inflammatory Drugs***			
Ketorolac	**Tab. 10 mg:** 20 mg sublingual for initial dose, 10 mg 4–6x/day (Max dose PO: 40 mg/day) **Ampoule 30 mg/mL:** 30 mg IV or 360 mg IM (Max dose IV: 120 mg/day)	Dyspnea, nausea, abdominal pain, diarrhea, headache, dizziness, liver enzyme alterations, drowsiness, tinnitus. Anaphylaxis, gastrointestinal bleeding, renal dysfunction, bronchospasms, drug rashes, agranulocytosis	*Absolute*: hypersensitivity, active peptic ulcer, cerebrovascular hemorrhage, third trimester of pregnancy or lactation
Ketoprofen	**Capsule 500–1000 mg:** 50 mg PO every 6 hrs or 100 mg PO every 12 hrs **Ampoule 100 mg:** 100 mg IV (dilute in 100 mL NS/D5W for IV infusion over 20 min)		*Absolute*: hypersensitivity, active peptic ulcer, gastrointestinal bleeding, third trimester of pregnancy or lactation
***Opioids***			
Tramadol	**Tab. 50–100 mg** **Ampoule 50 mg/mL (2 mL):** 50 to 100 mg PO, IV, or IM every 6 hrs (Max 400 mg/day) (dilute 1 mg/mL NS/D5W for slow infusion over 30 to 60 min)	Dizziness, drowsiness, sedation, headache, agitation, sweating, constipation, dyspepsia, nausea, vomiting, dry mouth, urinary retention, bradycardia, hyponatremia, seizures, bradycardia, miosis, respiratory depression (lower risk with Tramadol compared to other opioids)	*Absolute*: hypersensitivity, gastrointestinal obstruction including paralytic ileus, use of MAO inhibitors in the last 14 days *Relative*: elderly, respiratory depression, alcoholism. Use with caution in patients with liver or kidney failure
Meperidine	**Ampoule 50 mg/mL (2 mL):** 1 mg/kg IM (preferably) every 4 hrs (Max 500 mg/day) Initial dose of 25–50 mg IV (dilute in 10 mL NS/D5W slow or SC if necessary)		
Morphine	**Tab. 10 mg:** 5 to 30 mg PO every 4 hrs **Ampoule 10 mg/mL (1 mL)** **Ampoule 1 mg/mL (2 mL):** 1 mg/kg IM or IV every 4 hrs		
***Alpha Blockers***			
Tamsulosin	**Tab. 0.4 mg:** 0.4 mg PO once/day	Dizziness, orthostatic hypotension, ejaculation disorders, headache, rhinitis	*Absolute*: hypersensitivity, concurrent use with strong CYP3A4 inhibitors (e.g., ketoconazole)

### Summary and Recommendations

NSAIDs are effective in treating renal colic and superior to opioids. The use of opioids for the management of renal colic should be minimized (Grade of recommendation: A; Level of evidence: 1).

Intravenous hydration for the sole purpose of promoting the forced passage of stones should be avoided (Grade of recommendation: B; Level of evidence: 1).

The role of medical expulsive therapy (MET) in promoting the spontaneous passage of kidney stones is controversial, but current literature suggests that it is beneficial for distal ureteral stones sized between 5 and 10 mm. The advantages and disadvantages of MET should be discussed with the patient in a shared decision-making process (Grade of recommendation: A; Level of evidence: 1).

## Metabolic Investigation

### Who to Investigate?

The aim of laboratory diagnosis of stone-forming patients based on metabolic activity is to efficiently and cost-effectively identify distinct behavioral and pathophysiological differences for more effective therapeutic planning and prevention of lithiasis recurrence. The type and extent of the evaluation will depend on the severity of the condition; the distinction between single, recurrent, or occasionally recurrent stone formers; evidence of systemic disease and/or risk factors associated with recurrence; the presence of a family history of nephrolithiasis (NL); the patient’s interest in stone prevention, and finally the financing entity (SUS–National Health System of Brazil, health insurance, or personal resources).

By definition, single stone formers are patients with a single, solitary kidney stone episode, whether symptomatic or not. Recurrent nephrolithiasis is defined by two or more episodes of calculi, either synchronous or at different points in time. Occasionally recurrent stones involve the category of patients who experience recurrence at intervals greater than five years, and who could present as an intermediate risk category between single and recurrent stone formers. However, since metabolic abnormalities are similar in both single and recurrent formers, the complexity of the metabolic study should be guided by the aforementioned clinical characteristics or by cost-effectiveness^36,37^. Rule et al.^38^ estimated recurrence rates of 11%, 20%, 31%, and 39% at 2, 5, 10, and 15 years, respectively. They identified younger age, male sex, white ethnicity, family history, prior asymptomatic or non-obstructive stones, symptomatic pelvic or lower pole calculi on imaging, macroscopic hematuria, and uric acid stone composition as risk factors. These data may be used in a nomogram (ROKS nomogram) to estimate the risk of recurrence and subsequent shared diagnostic and therapeutic decision-making^38^. However, this approach has not been validated in other populations, particularly among Brazilians.

Another important distinction is between the *metabolically active* stone-former (new stone formation or stone growth on serial imaging, and/or clinical events related to nephrolithiasis during follow-up) and the *metabolically inactive* stone-former (no apparent stone activity on imaging and during clinical follow-up)^37^.

In line with international guidelines, it is recommended that recurrent stone formers and high-risk single stone formers (those with the risk factors listed above) or first-time stone formers interested in preventing future events should be referred for an individualized metabolic evaluation^39,40^.

Laboratory evaluation of NL patients is based on general serum tests, including biochemical and hormonal exams, also aimed at investigating electrolyte and acid-base disorders, simple urine or urine sediment tests (synonyms: urinalysis, UA test), and metabolic evaluation in 24-hour urine^37^. Laboratory assessments are used to guide the decision on initial treatment, also identifying the existence of other diagnoses of tubulopathies and endocrinopathies associated with NL and/or nephrocalcinosis (NC).

### How to Investigate?

#### Serum biochemistry

As shown in Table 3, the main tests to be requested are as follows. Reference values are merely a suggestion, as they vary according to each laboratory’s standardization and differ among populations, thus hindering comparisons with international guidelines. The rationale for these tests is outlined below:

**Table 3:** Serum laboratory parameters in adults

Serum parameters	Reference range	Relevance in diagnosis
Creatinine	0.6–1.0 mg/dL (F) 0.8–1.2 mg/dL (M)	Calculation of eGFR (estimated Glomerular Filtration Rate)
Sodium	135–145 mEq/L	Rule out hyponatremia
Potassium	3.0 –5.0 mEq/L	Influence on treatment – Hypokalemia (check for RTA, tubulopathies)
Calcium	8.6–10.2 mg/dL (total calcium)	**Elevated:** Primary hyperparathyroidism (always evaluate simultaneously with Parathormone (PTH)
	1.12–1.32 mmol/L (ionized calcium)	**Reduced:** Hypoparathyroidism
Uric Acid	4.6 mg/dL	**Elevated:** Gout, Metabolic syndrome **Reduced:** Xanthinuria
Chloride	98–112 mmol/L	Calculation of Anion Gap
Phosphorus	3.0–4.5 mg/dL	**Reduced:** Phosphate-wasting tubulopathies (calculate TmP)/Primary hyperparathyroidism **Elevated:** CKD or Hypoparathyroidism
Magnesium	1.6–2.6 mg/dL	Tubulopathies (Bartter, FHHNC)
Oxalate	<20.0–26.6 mol/L	Cases of recurrent NC or NL with GFR < 30 mL/min/1.73m^2^
Venous Blood Gas	pH: 7.35–7.45 pO_2_: 30–50 mmHg pCO_2_: 35–45 mmHg HCO_3_: 22–26 mmol/L BE: +2 mmol/L	Evaluate HCO3 for acidosis (RTA, etc.) or alkalosis (Bartter, etc.)
PTH	20–66 pg/mL (40–49 years) 21–71 pg/mL (50–59 years) 22–84 pg/mL (>59 years)	**Elevated:** Primary hyperparathyroidism (always evaluate simultaneously with calcium) **Reduced:** Hypoparathyroidism
25(OH)D_3_	>20 ng/mL (healthy population) >30 ng/mL (risk for bone loss)	Vitamin D deficiency Reduced bone density/1,25(OH)2D3 in genetic diseases

Abbreviations – RTA: renal tubular acidosis; PTH: parathormone; TmP: tubular maximum reabsorption of phosphate.

#### Creatinine

Measuring serum creatinine is essential for evaluating renal function in stone-forming patients, by calculating the estimated Glomerular Filtration Rate (eGFR) using the CKD-EPI 2021 equation. Chronic Kidney Disease (CKD) may cause secondary electrolyte abnormalities, complicating the diagnosis of metabolic alterations involved in nephrolithiasis or of primary tubulopathies related to renal stone disease. Conversely, NL can also progress to CKD. The main related risk factors are malformations of the urinary tract, stones composed of cystine, xanthine, or struvite, frequent urinary tract infections, overweight, female sex, single kidney, obstructive nephropathy, genetic disorders such as primary hyperoxaluria, renal tubular acidosis, or other hereditary tubulopathies with nephrocalcinosis.

#### Sodium (Na)

Determining serum Na is important for evaluating tubulopathies and for calculating the anion gap, which in turn is used in the diagnosis of acidosis. Additionally, a population-based study suggested that recent and persistent hyponatremia may also be associated with a higher occurrence of kidney stone disease^41^. In acute settings, hypernatremia may indicate volume contraction at the expense of free water loss.

#### Potassium (K)

As with sodium, determining serum K is important for assessing tubulopathies and acid-base disorders associated with NL, since hypokalemia is a common manifestation in monogenic diseases (e.g. Bartter syndrome or distal renal tubular acidosis, etc.).

#### Calcium (CA)

Elevated serum Ca may result in hypercalciuria, which is associated with an increased risk of kidney stone formation. The most common cause of hypercalcemia is primary hyperparathyroidism, characterized by the concomitant presence of elevated serum calcium and parathormone (PTH) levels. Monogenic diseases related to the metabolism of 25(OH)-hydroxyvitamin D, 25(OH)D, or 1,25-dihydroxyvitamin D, 1,25(OH)D, such as CYP24A1 hydroxylase deficiency, may present with hypercalcemia. Conversely, in primary or secondary hypoparathyroidism (following parathyroidectomy) and other monogenic disorders, severe and symptomatic hypocalcemia can manifest, accompanied by hypercalciuria, resulting in NL or NC. Changes in total Ca (adjusted for albumin) or in ionized calcium dosage make it imperative to evaluate the entire calcium metabolism axis for the investigation of the aforementioned conditions.

#### Uric acid

Hyperuricemia is common in patients with metabolic syndrome or diabetes mellitus, which in turn is associated with a low (acidic) urinary pH, leading to uric acid lithiasis. The diagnosis of gout should be considered, depending on a suggestive clinical history, particularly in patients with radiolucent stones or with uric acid composition in their physical/crystallographic analysis. On the other hand, very low uric acid levels may suggest monogenic diseases associated with NL, such as xanthinuria.

#### Chloride (Cl)

Chloride dosage, as well as Na and bicarbonate (HCO3), is useful for calculating the anion gap in the assessment of acid-base disorders linked to NL/NC. However, it is not among the first-line or extremely essential dosages in NL.

#### Phosphorus (P)

Hypophosphatemia as a risk factor for kidney stones in adults is more commonly observed in the context of primary hyperparathyroidism, but also denotes potential monogenic genetic disorders associated with phosphaturia, rickets, and nephrocalcinosis. When monogenic tubulopathies are suspected, serum P measurement is extremely important, in conjunction with its level measured in a spot urine sample (and with creatinine determination in the same sample), for calculating the fractional excretion of phosphorus and the maximum tubular transport of P.

#### Bicarbonate (HCO3)

Measurement of bicarbonate, ideally performed by venous blood gas analysis, is essential in the diagnosis of acid-base disorders related to both alkalosis (e.g. Bartter and Gitelman syndrome) and acidosis (e.g. in distal renal tubular acidosis [dRTA]) associated with NL or NC. It may also help in the supplementary diagnosis of complex tubulopathies. However, it should be noted that, in the case of distal RTA, the blood gas analysis should be performed on the same day as the measurement of 12-hour fast urinary pH (second micturition).

#### 25(OH)-vitamin D (25(OH)D)

In addition to evaluating vitamin D status, 25(OH)D measurement, when combined with Ca, P, and PTH, plays an important role in assessing bone metabolism, which is fundamental in the context of calcium nephrolithiasis. Changes in its levels could also help in the context of monogenic diseases involving vitamin D metabolism in association with NL, and sometimes complemented by dosages of its active metabolite, 1,25(OH)_2_D_3_.

#### Parathormone (PTH)

The association of NL with primary hyperpara­thyroidism (PHPT) should always be investigated when serum calcium is elevated (see above), but it can also increase secondarily in the context of reduced renal function and hypovitaminosis D, which should be excluded. PHPT is one of the most common causes of nephrocalcinosis in adults. Current reference values vary with age^42^, as shown in Table 3.

#### Magnesium (Mg)

Hypomagnesemia may be related to genetic tubulopathies associated with nephrolithiasis, such as in Bartter syndrome, but it may also suggest other monogenic diseases, such as familial hypomagnesemia with hypercalciuria and nephrocalcinosis (FHHNC), which is linked to cases of severe NC.

#### Oxalate

Serum oxalate should be measured in all patients with recurrent NL or NC, of early onset and with a positive family history, to rule out primary hyperoxaluria (PH1), when the eGFR is below 30mL/min/1.73m^2^, because in this case, urinary oxalate may not be elevated^43^.

#### 24-Hour urine biochemistry and per spot sample

The 24-hour urine assessment for a baseline metabolic profile should be obtained at least 3 to 8 weeks after the last stone clearance, whether spontaneous or surgical (provided the patient is without a double J catheter). Patients undergoing metabolic evaluation should have an unobstructed urinary tract, be on their usual diet, and have no urinary tract infection in their current therapeutic regimen, proven by urine culture. It is recommended, according to the latest guidelines, that at least two 24-hour urine samples be considered for diagnosis, under the usual diet, collected on 2 non-consecutive days for a metabolic assessment in recurrent stone formers, single stone formers with a high risk of recurrence, individuals with a single kidney, or in cases of NC^44,45,46^. All the analytes listed further can and should (for comparisons between the analytes – for example Na and Ca) be measured in the same sample, without the need for preservatives (acid or alkaline) in the containers. This reduces the inconvenience and cost of more than two 24-hour urine collections per patient^44,47^ and eliminate the interference with pH measurement in 24-hour urine^47^. Preservatives could be added post-collection, in the laboratory, without compromising the results^48,49^. To prevent urine contamination and its interference with citrate dosage, thymol or boric acid can be added afterwards^44,49^. Within 3 to 6 months of starting treatment, a new 24-hour urine collection may be requested to assess response to dietary and/or pharmacological therapy and monitor adverse events^37, 39^. Subsequently, follow-up may be annual, to assess the effectiveness and adherence to therapy. Nevertheless, as highlighted by Fink et al.^39^, there is no definitive evidence from clinical trials showing that the normalization of follow-up biochemical tests is a valid surrogate for predicting a reduction in stone recurrence. Therefore, follow-up should be individualized according to possible underlying etiologies. The risk factors considered are early onset of NL, family history, recurrence, brushite (calcium phosphate) stones, uric acid, or those related to urinary tract infection (struvite)^46^. In addition to these, anatomical abnormalities of the urinary tract, systemic or genetic diseases, and environmental factors also contribute to increasing the risk of recurrence. The main analytes to be evaluated in 24-hour urine are shown in Table 4. Reference values are merely a suggestion, as they vary according to each laboratory’s standardization and also differ among populations, thus hindering comparisons with international guide­lines. Attention and action levels have been indicated for different cutoff values for the various parameters. Obviously, the risk of NL recurrence should be considered individually for each patient. Therapeutic decisions could be applied from the attention level and are recommended from the action level.

**Table 4:** Urinary laboratory parameters in adults

24-hour urine analytes	Reference range	Attention level	Action level	Relevance
Volume	>2.0–2.5 L/day (25–30 mL/kg/day)	<1.5 L/day	<1 L/day	Adequacy of fluid intake
Creatinine	Adults < 50 years 14–20 mg/kg/day (F) 20–25 mg/kg/day (M)	–	–	Adequacy of urine collection
Calcium	<150 mg/day (varies with diet)	150–200 mg	>250 mg (F) >300 mg (M) >4 mg/kg/day (F/M)	Risk of stone formation is continuous with calciuria
Uric Acid	250–750 mg/day	–	>750 mg (F) >800 mg (M)	Evaluate along with urinary pH
Oxalate	<45 mg/day (eGFR > 60 mL/min/1.73m^2^)	4–31 mg (F) 7–44 mg (M)	>40 mg (F) >45 mg (M)	>90 mg/day (1.0 mmol/day) suggests Primary Hyperoxaluria (HP1)
Citrate	>320 mg/day	320–450 mg/day	<320 mg/day	Hypocitraturia or distal RTA
Sodium	50–100 mEq/day	100–150 mEq	150 mEq	Adequacy of salt intake
pH	*24-hour urine* (no reference value)	Varies with diet	<5.5 U24hs (Uric acid lithiasis?) >7.0 U24hs (Calcium Phosphate, Struvite?)	–
	*12-hour fasting spot urine sample* (2nd void) pH < 5.8	–	pH > 5.8 and 6.2 (U24h) (exclude distal RTA)	Evaluate along with plasma HCO3
Phosphorus	400–1300 mg/day	Varies with diet	–	FEP% + TmP: (best in spot urine sample)
Potassium	20–100 mEq/day	Varies with diet	–	TTKG (transtubular K gradient)
Urea	15–40 g/day	–	–	Adequacy of protein intake
Magnesium	1.3–1.7 mg/kg/day	–	–	Low evidence
Chloride	110–250 mEq/day	–	–	Anion Gap (urine)
Cystine	6.7–27.6 mg/day 28–115 μmol/day)	>0.8 mmoL/day	–	Cystinuria
Ammonia	<50 mmol/day	–	–	Usually not available
Sulfate	20–80 mmol/day	–	–	Usually not available

In a nutshell, urinary volume indicates the adequacy of fluid intake, while urinary creatinine excretion provides information on the adequacy of collection. The determination of calciuria in 24-hour urine is important in the diagnosis of idiopathic hypercalciuria, or of monogenic causes, or associated with distal RTA. However, the risk of lithiasis is continuous with calciuria, with no cut-off values^50^, as with other analytes^51^. There is still no consensus regarding the potential replacement by calcium measurement in spot urines to establish these diagnoses in adults^52^. Uricosuria is only of current importance in the context of uric stone disease if considered in conjunction with urinary pH in a 24-hour urine sample. Urinary oxalate should be measured in every patient with NL or NC to help differentiate between hyperoxaluria of secondary or primary cause. In the latter case, if the patient has an eGFR < 30 mL/min/1.73m^2^, plasma oxalate measurement should be performed as previously mentioned^43^. Recently, elevated 24-hour oxaluria levels, outside the context of PH1, have been linked to progression to CKD^53^. Citrate measurement is important for diagnosing idiopathic hypocitraturia or hypocitraturia associated with acidosis, such as in distal RTA. Urinary sodium reflects salt intake. The pH in 24-hour urine has no reference value and varies according to whether the diet is acidogenic or not. Low pH values suggest uric acid stones, related to obesity, while high values are related to calcium phosphate or struvite stones. The pH values obtained in urinary sediment are only relevant when reduced (less than 5.5), as they rule out distal RTA, since higher values, in the alkaline range, may result from bacterial contamination. For the diagnosis of distal RTA, a spot urine sample should be collected after a 12-hour fast, obtained at the second voiding, once the fasting period has been completed. The test should be conducted on the same day as plasma HCO3 measurement, accessing the acidosis state, for the diagnosis of distal RTA in its complete form. In the case of normal HCO3 levels, the NH4Cl (ammonium chloride) loading test is indicated to complement the diagnosis of incomplete forms of distal RTA. Phosphorus measurement in 24-hour urine indirectly reflects protein intake and is used in some crystallization indices. Despite its usefulness in tubulopathies with phosphaturia and hypophosphatemic rickets, the ideal approach in phosphaturia is to calculate the fractional excretion of phosphorus (FeP%), collected alongside creatinine in spot urine and serum using the following formula: FeP = [P (Urine) × Creatinine (Serum)]/[P (Serum) × Creatinine (Urine)] × 100 and the calculation of tubular reabsorption of phosphorus [1 – FeP] × 100. Similarly, potassium measurement can reflect potassium intake, but in cases of hypokalemia, which may occur in tubulopathies associated with nephrolithiasis and/or nephrocalcinosis, calculating the TTKG (transtubular K gradient) differentiates hypokalemia due to renal loss (>4) from that of gastrointestinal origin (<2) using the formula: [K (Urine) × Osm (Serum)/K (Serum) × Osm (Urine). Urea measurement is useful for protein assessment calculated using the formula for the Protein Equivalent of Urinary Nitrogen Appearance (PNA), as follows: PNA = (urinary urea nitrogen + [0.031 × weight]) × 6.25, where urinary urea nitrogen is equal to (urinary urea/2.14 × urinary volume). However, it should be emphasized that the PNA reflects total protein intake, and that only the determination of urinary sulphate indicates animal protein intake. The measurement of urinary magnesium has little evidence of utility in the evaluation of patients with nephrolithiasis, and its implication in therapy has been questioned in the only clinical study using magnesium hydroxide. Its importance lies in its inclusion in urinary crystallization formulas, such as the Tiselius formula^54^. Chloride has limited utility in isolation, but it helps to calculate the urinary anion gap, defined by urinary chloride concentrations subtracted from the sum of Na + K, used as an indirect measure of urinary ammonium (NH4)^55^. The latter is scarcely available in laboratories, although it is very useful in diagnosing acidosis states with a normal anion gap. The presence of cystine may be qualitatively determined by the sodium nitroprusside colorimetric test and complemented by the quantitative determination of cystine, which is essential for the diagnosis of cystinuria. Sulphate measurement shows a good correlation with protein intake; however, it is unavailable in our setting, and the need for its determination remains controversial.

Finally, despite the multifactorial etiology, with the advent of new sequencing techniques, monogenic causes of nephrolithiasis should also be investigated. These causes may be present in up to 30% of children and 10% of adults, with more than 35 different genes currently implicated^56^.

**Figure 3:**
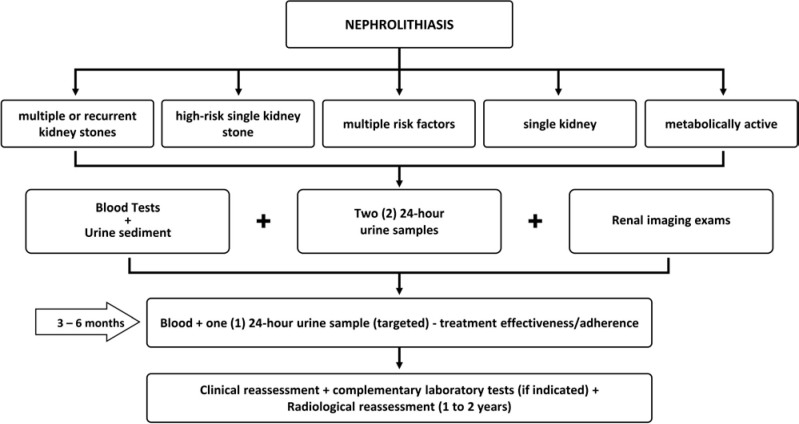
Investigation and follow-up of patients with nephrolithiasis.

Figure 3 summarizes the steps in the investigation and follow-up of nephrolithiasis patients.

### Summary and Recommendations

It is recommended, in accordance with international guidelines, that recurrent, bilateral or multiple stone formers, single but high-risk stone formers, patients with a single kidney, metabolically active patients, or those with multiple risk factors undergo a complete and individualized metabolic evaluation. The main risk factors considered are the onset of nephrolithiasis and/or nephrocalcinosis at an early age, family history, high recurrence rates, brushite stones (calcium phosphate), uric acid stones, or infection-related stones (struvite) (Grade of recommendation B; Level of evidence 2).

The metabolic evaluation should comprise blood samples, spot urine sample, and 24-hour urine collections. Serum tests should at least include creatinine, Na, K, uric acid, HCO3, total calcium + albumin or ionized calcium, and PTH measurements. Spot urine samples should include urinary sediment and fasting pH. In 24-hour urine, the minimum parameters to be analyzed involve determining the volume, creatinine, Ca, Na, K, oxalate, citrate, uric acid, and pH. The qualitative measurement of cystine may indicate its quantitative determination *a posteriori* (Grade of recommendation B; Level of evidence 2).

It is ideally recommended that at least two 24-hour urine samples be considered for diagnosis, under the usual diet, collected on 2 non-consecutive days. The baseline metabolic profile should be obtained at least 3 to 8 weeks after the last stone clearance, whether spontaneous or surgical, in the absence of a urinary tract infection proven by urine culture (Grade of recommendation B; Level of evidence 2).

## Urinary Stone Analysis

The eliminated stone should be recovered and submitted for analysis whenever possible. Knowing the composition of the urinary stone is important for understanding the pathophysiology of the disease, preventing recurrence, and deciding on the clinical and/or surgical treatment modality. Brushite (dicalcium phosphate dihydrate), calcium oxalate monohydrate (COM), or cystine stones are known to be more resistant to fragmentation^40,44,57,58,59^. The collection can be performed directly by the urologist, during surgical removal, or by the patient him/herself. The patient should be instructed to filter the urine in the first few days following a pain crisis, and in the event of elimination of the stone or its fragments, to keep the material in a dry, clean container until it can be sent for analysis^60^.

### Chemical Analysis

Although it is the most commonly used technique in our setting, the chemical analysis of urinary stones only identifies the presence of individual ions and radicals, without being able to quantify or differentiate specific components, such as purine stones or those resulting from medication, for example, or even associations of components, currently being considered obsolete^60^.

### Crystallographic Analysis

Infrared spectroscopy and X-ray diffraction are more accurate qualitative and quantitative methods for determining the composition of urinary stones; they are therefore considered the gold standard. Physical methods are typically preceded by a microscopic (stereomicroscopic) analysis to morphologically characterize the stone, which depends on specialized training^61^. Infrared spectroscopy uses infrared radiation to induce atomic vibrations and analyzes the energy bands that emerge in the spectrum. Its drawbacks include limited availability in our region, high costs, and the fact that it is not yet included in the list of exams performed by the Public Health Service and Health Insurance Plans. X-ray diffraction is a technique that uses monochromatic rays to identify the constituents of kidney stones based on the unique diffraction patterns produced by the crystalline material, resulting in a characteristic diffractogram. Although it is an accurate technique, it is not widely accessible for regular clinical use due to its complexity and high cost^60,62,63^.

### Recurrence

Stone analysis may be repeated in cases of recurrent lithiasis, despite pharmacological therapy; when there is early recurrence, after complete removal of the stone; and in late recurrence, following a long period without lithogenesis, as the composition of the stone may change (changes in stone composition are reported in up to 21% of cases)^64^.

### Summary and Recommendations

Stones collected by patients or removed during surgical intervention should be sent for morphological analysis associated with a physical or crystallographic method, ideally using infrared spectroscopy or X-ray diffraction (Grade of recommendation C; Level of evidence: 3).

It is suggested to repeat the analysis of the calculus in patients with recurrent stones despite pharmacological therapy; in early recurrence after complete removal of the calculus; and in late recurrence after a long period without stones (Grade of recommendation C; Level of evidence 3).

## Radiological Investigation and Monitoring

### Imaging of the Urinary Tract

Non-contrast computed tomography (CT) is the most accurate imaging technique for identifying urinary stones due to its high sensitivity and specificity, as well as its ability to accurately measure stone size and assess other pathologies unrelated to stones^65^.

Low-dose CT has many of the same advantages as standard CT and reduces radiation exposure; however, its diagnostic accuracy is reduced in obese patients, and dose modulation should be considered^66^.

CT can also provide information on the composition of kidney stones. There is an attenuation measure, the Hounsfield Units (HU), which describes the density of tissues and substances. On a scale, water has a density of 0 (zero) HU, while bone density is 1000 HU. Uric acid stones have a density ranging between 200 and 400 HU, while calcium oxalate stones have a density between 600 and 1200 HU (2). More recently, the use of dual-energy CT has proven superior to conventional CT in differentiating between uric acid stones and calcium-based stones, with some studies reporting sensitivities and specificities close to >95%^67^.

In adults, compared to non-contrast CT (the reference exam, with sensitivity >95%), the sensitivity of ultrasonography was 60% and its specificity 90%. However, when these imaging modalities were subjected to a randomized clinical trial, they were found to have equivalent diagnostic accuracy in the context of the emergency department^17^.

Plain radiography of the kidney, ureter and bladder (KUB) is more useful in assessing the interval growth of stones in patients with known stone disease and has less utility in detecting stones in acute crisis, in emergency care (sensitivity of 58% and specificity of 90%), when compared to CT scanning^66^.

The sensitivity of MRI is variable, around 82%. And as with ultrasound, it may be higher due to the presence of associated hydronephrosis. When stones are visualized on MRI, the specificity is high (98.3%). In general, MRI is more expensive than a CT scan, as well as having significantly longer image acquisition times^66^.

During pregnancy, ultrasound may be used as a diagnostic tool, when necessary. However, physiological changes during pregnancy can mimic ureteral obstruction. Magnetic resonance imaging may be used as a second option to define the level of obstruction and visualize stones (which appear as a filling defect)^68^.

Symptomatic recurrence of a stone can range from minimal discomfort with the spontaneous passage of the stone to severe pain^69^. Radiographic recurrence, including new stone formation and the growth of existing stones, may be a useful and practical surrogate for symptomatic recurrence. Regrettably, the resolution of symptoms and the patient’s reports of successful urinary stone passage are not always confirmatory^70^.

There are no studies evaluating the frequency at which imaging diagnoses should be repeated in patients who have or have had kidney or ureteral stones. Based on consensus among specialists, it is suggested that patients be stratified^69^.

In a systematic review and meta-analysis conducted by the European Association of Urology with 5467 patients who underwent intervention and were “stone-free”, it was estimated that, for a safety margin of 80%, patients should undergo follow-up imaging examinations for at least 2 years (for patients with radiopaque stones) and for at least 3 years (for patients with radiolucent stones). For a 90% margin, follow-up should be conducted for 5 years in patients who have not experienced recurrence of stones^71^.

Patients with “residual fragments” with fragments <4 mm should undergo follow-up imaging for 4 years, since, over a 29-month period, the rates of need for urological reintervention in this group ranged from 17% to 29%, disease progression from 9% to 34%, and spontaneous passage of stones from 21% to 34%. In patients with fragments >4 mm, a new definitive urological intervention should be offered, given that the success rate is high (24% to 100%)^71^.

There is insufficient data for “patients with metabolic abnormalities – high risk”. According to consensus guidelines established following extensive discussion by a panel of experts from the European Society of Urology, less than 40% of patients with metabolic abnormalities without targeted pharmacological treatment remained stone-free over a 3-year follow-up period. Therefore, this follow-up period is proposed for this group of patients^71^.

**Figure 4:**
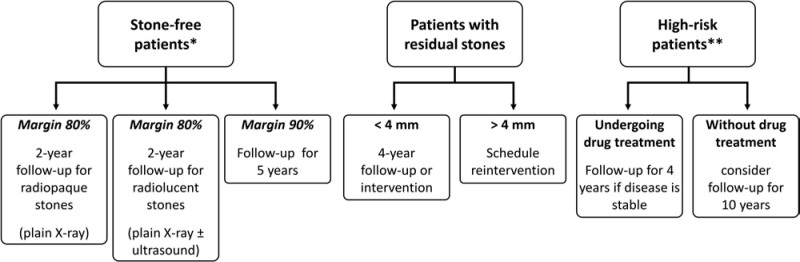
Recommendation for radiological follow-up of patients diagnosed with asymptomatic urinary stone disease.

Figure 4 summarizes the suggested follow-up for these various clinical situations.

Notes – *Stone-free: no fragments on post-operative imaging; **High risk: identified biochemical abnormality (hypercalciuria, hypocitraturia, hyperuricosuria, renal tubular acidosis) or high-risk stones, such as struvite or cystine.

### Summary and Recommendations

The resolution of symptoms, as well as patient reports regarding the passage of urinary stones, are not always confirmatory (Grade of recommendation: D; Level of evidence: 4).

Follow-up imaging is recommended to confirm stone passage (Grade of recommendation: B; Level of evidence: 2).

The imaging tests proposed for follow-up include plain abdominal X-ray and ultrasound, based on the characteristics of the stones and the preferences of the attending physician (Grade of recommendation: B; Level of evidence: 2).

Computed tomography should be reserved for symptomatic patients or those undergoing preoperative evaluation, to minimize radiation exposure (Grade of recommendation: C; Level of evidence: 3).

### Investigation of Mineral and Bone Disorders in Patients with Kidney Stones

Nephrolithiasis is associated with an increased risk of reduced bone mass. Several studies have reported decreased bone mineral density (BMD) in nephrolithiasis patients when compared to healthy individuals, along with an increased risk of fractures^72,73,74,75,76,77^. Bone demineralization may be related, among other factors, to lower calcium intake and higher urinary calcium levels. However, it has also been observed in normocalciuric or kidney stones patients not categorized according to urinary calcium excretion^78,79,80,81,82,83^.

A meta-analysis of 28 case-control studies compared 1595 patients with nephrolithiasis (LIT) to 3402 healthy control subjects (CONT) in their fourth decade of life by analyzing the standardized mean difference (SMD). It was shown that LIT patients had lower T-score values in the lumbar spine (in 7 studies, SMD = -0.69), total hip (in 7 studies, SMD = -0.82), and femoral neck (in 6 studies, SMD = -0.67). Regarding the risk of fractures, the analysis of data from 4 case-control studies has demonstrated a significant increase in the risk of fractures among LITs (Odds ratio, OR = 1.15). This trend was also observed in 2 longitudinal studies (OR = 1.31). In addition, the LIT group had a 4-fold higher risk of developing osteoporosis compared to the CONT group (OR = 4.12)^84^.

In the metabolic evaluation of patient with kidney stones, it is suggested to investigate underlying conditions or diseases that are more frequently associated with mineral and bone disorders, such as idiopathic hypercalciuria, distal renal tubular acidosis (complete or incomplete), nephrocalcinosis and/or medullary sponge kidney, primary hyperparathyroidism, malabsorption syndromes or individuals undergoing bariatric surgery, tubulopathies, or monogenic diseases^56^.

Dietary recommendations (adequate protein, sodium and calcium intake), vitamin D replacement, and pharmacological therapies indicated to reduce kidney stone formation (thiazide, citrate, or alkali) may contribute to stabilizing bone loss^36^.

Note: The reference standard for the diagnosis of osteoporosis, as defined by the World Health Organization (WHO), corresponds to a T-score ≤ 2.5 at the femoral neck (T-score was calculated in white women aged 20–29, NHANES database). Osteoporosis can be diagnosed in postmenopausal women and in men aged 50 and over if the T-score is ≤ 2.5 in the lumbar spine, femoral neck, or total hip. According to the new diagnostic criteria established by the WHO, the term “osteopenia” should no longer be used and should instead be replaced by “low bone mass” or “low BMD”, since this finding does not necessarily indicate an increased risk of fracture. In premenopausal women, children, and men under 50 years of age, the Z-score (rather than the T-score) should be used: Z-score ≤ 2.0 is defined as low bone mass for chronological age^85^.

### Summary and Recommendations

Individuals with nephrolithiasis are at increased risk of metabolic bone disease and fractures (Grade of recommendation B; Level of evidence 2). In the metabolic evaluation of patients with kidney stones, it is suggested to investigate underlying conditions or diseases more commonly associated with mineral and bone disorders (Grade of recommendation: C; Level of evidence: 3).

Kidney stone patients, particularly those with hypercalciuria, should be assessed for the risk of osteoporosis or bone mass loss through bone densitometry using dual-energy X-ray absorptiometry (DEXA)* (Grade of recommendation: B; Level of evidence: 2).

## Treatment

### Nutritional Recommendations

Diet plays a central role in the genesis and prevention of kidney stones. Although pharmacological treatment may be necessary for some patients, nutritional intervention may be sufficient to reduce the risk of urinary stone disease in many patients. There is no single specialized diet for the prevention of kidney stones, but several recommendations and principles are supported by intervention and epidemiological, or observational studies.

Table 5 summarizes the general nutritional guidelines for patients with kidney stones, as well as those related to specific metabolic disorders associated with nephrolithiasis.

**Table 5:** Nutritional recommendations according to risk factors and metabolic disorders

Risk factors/metabolic disorders	Nutritional recommendation	Recommendation level	References
Obesity	Adequacy of Body Mass Index (BMI)	A	86,87,88
	Balanced caloric reduction, appropriate to the patient's weight loss needs	D	
	Protein intake: 0.8 to 1.0 g/kg of ideal body weight/day (avoid high-protein diets like low-carb with potential acidogenic effects)		
Metabolic Syndrome (MS)/Type 2 Diabetes/Obesity/Hypertension/Dyslipidemia/Hyperuricemia	Dietary recommendation focusing on MS diagnostic criteria for the patient	D	89 90,91
	Caloric reduction with adequate animal protein/reduction of cholesterol-rich foods/saturated fats and high glycemic index foods/↓ Salt intake (NaCl) <6 g/day ↑↑ consumption of vegetables and fruits (alkaline diet)		
Bariatric Surgery	Calcium intake: 1000 to 1200 mg/day (consider supplementation*)	D	92
	↓ Intake of foods rich in oxalate; ↓ Salt intake (NaCl) <6 g/day		
	↓ Fat intake: 25–30% of total energy value of the diet		
	↑ Fluid intake (30 mL/kg/day + 400 mL insensible losses): urine output 2.5 to 3.0 L/day		
	Protein intake: 0.8 to 1.0 g/kg of ideal body weight/day		
	↑ Consumption of vegetables and fruits		
Low Urinary Volume	↑ Fluid intake (30 mL/kg/day + 400 mL insensible losses): urine output 2.5 to 3.0 L/day	A	93,94,95,96
Hypercalciuria	↓ Salt intake (NaCl) <6 g/day	A	96,97,98,99,100
	Protein intake: 0.8 to 1.0 g/kg of ideal body weight/day		
	↑ Consumption of vegetables and fruits / BMI adequacy		
	Calcium intake: 1000 to 1200 mg/day*	D	
Secondary Hyperoxaluria	Calcium intake: 1000 to 1200 mg/day*	A	97,101,102,103,104
	↓ Intake of foods rich in oxalate	B	
	Protein intake: 0.8 to 1.0 g/kg of ideal body weight/day	D	
Acidic Urinary pH/Hyperuricosuria	↑ Fluid intake (30 mL/kg/day + 400 mL insensible losses): urine output 2.5 to 3.0 L/day		
	Protein intake: 0.8 to 1.0 g/kg of ideal body weight/day with ↓ animal protein, ↑ consumption of vegetables and fruits (alkaline diet) – DASH diet pattern	D	97,105,106,107,108
	BMI adequacy (see obesity recommendation above)		
	Note: No beneficial evidence for ↓ purines in uric acid lithiasis/↓ alcohol consumption, especially beer, for gout^109^		
Hypocitraturia	Protein intake: 0.8 to 1.0 g/kg of ideal body weight/day with ↓ animal protein, ↑ consumption of vegetables and fruits (alkaline diet) – DASH diet pattern	A	98,105, 110,111,112,113
	↑ Consumption of foods rich in citrate/malate (e.g., lemon, orange, tangerine, melon)	B	
Cystinuria	↑ Fluid intake for urine output ≥ 3.0 L/24h and ↓ cystine concentration <250 mg/L, consume vegetables and fruits (alkaline diet) with DASH diet pattern	D	114,115,116
	Protein intake: 0.8 to 1.0 g/kg of ideal body weight/day/Salt intake: <6 g/day		
Lithiasis without Identified Metabolic Disorder	↑ Fluid intake (30 mL/kg/day + 400 mL insensible losses): urine output 2.5 to 3.0 L/day	A	86 87,88,94, 105,117,118,119,120,121,122,123,124,,125
	Adherence to a DASH or Mediterranean diet pattern		
	Calcium intake: 1000 to 1200 mg/day		
	Protein intake: 0.8 to 1.0 g/kg of ideal body weight/day		
	↓ Salt intake (NaCl): <6 g/day		
	↑ Consumption of vegetables and fruits		
	BMI adequacy/↓ foods rich in oxalate (controversial)	A/D	

#### Dietary supplements, vitamins, and probiotics

Calcium supplements, unlike dietary calcium, may be associated with up to a 20% increase in the risk of developing kidney stones. This occurs due to its intake at times other than meals, which does not have a significant chelating effect on oxalate, or due to the accumulation of calcium supplementation in addition to dietary calcium intake^121^. It is therefore recommended to prioritize improving diet by increasing consumption of calcium-rich foods (Grade of Recommendation **D**). The consumption of vitamin C supplements in doses greater than 1000 mg per day has been linked to a 16% increase in the occurrence of kidney stones, as well as to increases in urinary oxalate levels in men^126^. Intervention studies with doses of 1000 and 2000 mg per day have also revealed increases of up to 61% in oxaluria levels^127,128^. It is therefore advisable to avoid the use of supplements (Grade of Recommendation **D**). Vitamin D supplementation may increase calcium excretion and the risk of nephrolithiasis^129, 130^. However, studies in this area show divergent results^131,132,133^. Consequently, it is essential to evaluate vitamin D supplementation in kidney stone patients on a personalized basis, considering individual needs, serum calcium levels, and the effect on urinary calcium (Grade of Recommendation **D**). Stone-forming individuals appear to have reduced gut microbial diversity, including lower colonization by *Oxalobacter formigenes*, which plays a crucial role in the degradation of dietary oxalate^134^. Despite the possible link with reduced urinary oxalate levels, *O. formigenes* supplementation has not conclusively demonstrated this beneficial effect^135,136^. In addition to *Oxalobacter formigenes*, strains of *Lactobacillus* and *Bifidobacterium* also have the ability to metabolize dietary oxalate, which would limit its absorption and subsequent urinary excretion^137^. However, the results of randomized clinical studies have been contradictory^138,139,140,141^. Individualized prescription may be considered as an alternative and adjuvant approach, which requires further investigation regarding dosage, type, and duration of administration (Grade of Recommendation **D**). Protein supplements are widely used to build muscle mass and improve performance during physical exercise. The consum­ption of whey protein or albumin by healthy individuals, over a short period and under a controlled diet, showed no significant changes in factors related to lithogenesis^142^. However, considering the current recommendations for adopting a diet with normal protein levels for patients with nephrolithiasis, it is crucial to evaluate the prescription of protein supplements on an individual basis, considering specific needs and urinary biochemical monitoring (Grade of Recommendation **D**).

### Summary and Recommendations

Dietary recommendations, in general, include adequate fluid intake, moderate calcium consumption, low sodium content, and restriction of animal proteins in the diet (Grade of recommendation B; Level of evidence: 2).

Recommendations can be targeted to the identified metabolic profile, but weight adequacy and adherence to a DASH or Mediterranean diet pattern are useful regardless of these (Grade of recommendation B; Level of evidence: 2).

The effects of prescribing supplements and vitamins remain controversial and should be tested and analyzed on an individual basis (Grade of recommendation D; Level of evidence: 4).

### Pharmacological Treatment

#### Thiazides

Thiazide diuretics should be offered to patients with high urinary calcium concentration and/or recurrence of calcium urinary stone disease. Although the precise mechanism of action of thiazide or thiazide-like drugs (chlorthalidone; indapamide) has not been fully elucidated, they would be appropriate when dietary measures and increased fluid intake are not sufficient to control recurrence of kidney stones and in cases of bone mass loss^143^. In these circumstances, these drugs are considered appropriate for both calcium oxalate and calcium phosphate stones.

Although the effects of the different thiazides are relatively similar in terms of preventing stone recurrence, their potency and side effects may differ^144^. However, there is a lack of more robust clinical studies which include hypercalciuric patients aimed at comparing potency and side effects, using different doses and with long-term follow-up^145^. Suggested doses and regimens include hydrochlorothiazide 25 mg twice daily or 50 mg daily, chlorthalidone 25–50 mg once daily, or indapamide SR 1.5 mg once daily^144^.

It is strongly recommended to monitor serum potassium levels when using these drugs to avoid increased cardiovascular risk and minimize glucose intolerance, among other side effects. Generally, the combination of drugs, such as potassium citrate or amiloride, can minimize hypokalemia. In general terms, hydrochlorothiazide has the shortest half-life, around 8–12 hours, which would require administration every 12 hours to maintain control of calciuria throughout the 24 hours. However, using diuretics at night would not be as appropriate, due to the potential impact on sleep quality and the risk of falls, especially in the elderly. From this perspective, chlorthalidone or indapamide (unfortunately not available through the Brazilian public healthcare system – SUS) would be more appropriate, as they have a longer half-life, around 24–36 hours.

A highly debated potential side effect observed after long-term use of thiazides or thiazide-like drugs is their association with an increased risk of skin cancer, especially squamous cell carcinoma of the skin and lips, due to their photosensitizing property^143,146^.

Several clinical trials have already demonstrated, albeit with small numbers of patients, that thiazides are effective in preventing the recurrence of kidney stones. However, the role of this class of drugs has recently been questioned with the publication of the Swiss NOSTONE study, which randomized 416 patients with recurrent calcium nephrolithiasis to receive hydrochlorothiazide (in daily doses of 12.5 mg, 25 mg, or 50 mg) or placebo^147^. The primary outcome was a composite of symptomatic or radiological recurrence of kidney stones, with a median follow-up duration of 2.9 years. Results showed no statistically significant differences in the primary outcome between hydrochlorothiazide at any dose and placebo, raising significant controversy about whether thiazides should remain in the stone prevention arsenal. However, several points may have biased NOSTONE towards a null result. These include the use of a shorter acting thiazide administered once daily, dietary variations that potentially affect the medication’s effect, not including only patients with hypercalciuria, the relatively short duration of follow-up and the primary outcome considering the passage of existing stones as a new stone event^148^. In a *post hoc* analysis, when considering exclusively the formation or growth of new stones as the primary outcome, the two groups receiving the highest doses of hydrochlorothiazide showed significant reductions in the risk of stone formation^149^.

#### Sodium bicarbonate and potassium citrate

Bicarbonate, citrate salts, and other metabolites of the citric acid cycle generate an alkaline load in the body. The action of bicarbonate is immediate, as it neutralizes gastric acid, and when present in excess in relation to acid secretion, it is absorbed^150^. Citrate is metabolized as citric acid with the uptake of a proton, leading to the formation of new bicarbonate in the blood. The renal proximal tubule cells can excrete the excess alkali as bicarbonate, increasing the urine pH, or as citrate, which does not alter the urinary pH. The latter occurs because the reabsorption of filtered citrate is reduced by the alkaline load^151^. The alkaline load also reduces calcium in the urine, increasing calcium reabsorption in the renal tubules. However, this is only true for potassium salts; sodium alkali may not be as effective.

Both sodium and potassium alkali salts can effectively increase urinary citrate levels; however, potassium citrate is generally preferred over sodium citrate. This preference is due to sodium’s potential to increase urinary calcium levels, which may lead to increased calciuria^152^.

Potassium citrate is an adjuvant medication in the treatment of hypercalciuria, as it reduces urinary saturation by complexing with calcium both in the intestine and possibly in the kidneys^153^. Additionally, citrate increases the dissociation of uric acid, reducing the likelihood of its precipitation and preventing the formation of mixed stones with calcium oxalate. Evidence in the literature further suggests that alkali­nizing agents inhibit bone resorption, improve bone mass, induce hypocalciuria, and stimulate osteoblastic activity^151^. The daily therapeutic dose ranges from 20 to 60 mEq, according to each patient’s tolerance. In special cases, such as patients with cystinuria, the dose may be increased up to 90 mEq/day.

In our country, potassium citrate is the most widely available and recommended. It can be found in ready-made formulations or in compounding pharmacies. It is usually available in 5, 10, and 15 mEq slow-release tablets or capsules.

A major limitation to the use of this medication is gastric intolerance and gastroesophageal reflux disease, which can be minimized by taking it after meals^154^. Special attention should be paid to patients with chronic kidney disease due to the risk of hyperkalemia or metabolic alkalosis, especially if associated with the use of other alkalis, such as sodium bicarbonate.

#### Allopurinol

Allopurinol is a medication used to prevent nephrolithiasis in patients with calcium oxalate or uric acid stones, especially when associated with hyperuricosuria (urinary excretion of uric acid >800 mg/day)^155^. However, in the latter situation, urinary pH should be concomitantly maintained alkaline to achieve greater solubility of uric acid.

Allopurinol acts as a xanthine oxidase inhibitor, an enzyme that converts hypoxanthine into xanthine and, subsequently, xanthine into uric acid. By inhibiting this enzyme, allopurinol reduces uric acid production, resulting in lower concentrations of uric acid in both blood and urine^156^. Benefits in preventing kidney stones include reducing uric acid saturation, which decreases the formation of uric acid crystals that may act as a nucleus for stone formation. In addition, uric acid can form crystals that serve as a nucleus for calcium oxalate precipitation. By reducing the concentration of uric acid, allopurinol may also help prevent the formation of calcium oxalate stones^157^.

Ettinger et al., in 1986, randomized 60 patients with recurrent nephrolithiasis, hyperuricosuria, and normocalciuria, to receive allopurinol (100 mg three times a day) or placebo. After the study, the placebo group showed a 63.4% reduction in stones (P < 0.001), while the allopurinol group had an 81.2% reduction in stones (P < 0.001). During the study period, the average rate of stone events was 0.26 per patient per year in the placebo group and 0.12 in the allopurinol group^158^.

Allopurinol dosage is usually individualized based on serum and urinary uric acid levels, as well as the patient’s renal function. The typical dose ranges from 100 to 300 mg per day and can be adjusted as required. Adverse effects may include skin rashes, gastrointestinal disorders, and, rarely, serious hypersensitivity reactions such as Stevens-Johnson syndrome^159^.

#### Gliflozins

Gliflozins, also known as sodium-glucose cotransporter inhibitors (SGLT2 inhibitors), are drugs that act on the kidneys, preventing glucose reabsorption in the renal tubules, and promoting the excretion of glucose in the urine. These drugs have demonstrated in several studies a significant reduction in cardiovascular and renal outcomes in both diabetic and non-diabetic patients^160^.

In studies not specifically designed to evaluate the effect of gliflozins, an increase in urinary volume, higher citraturia, decreased urinary pH, anti-inflammatory and antifibrotic effects, with suppression of macrophage marker expression (*in vivo* and *in vitro* studies), decreased serum uric acid, with increased uricosuria, and reduced urinary supersaturation for calcium phosphate were noted in healthy subjects^161,162^.

Although there are encouraging preliminary results with SGLT2 inhibitors, these drugs need to be tested in patients with nephrolithiasis, with a primary focus on reducing urinary stones. A viable approach at this point would be to consider the association of gliflozins in patients with kidney stones who already have established indications for these drugs, such as in the presence of diabetic kidney disease, for example^163^.

#### D-penicillamine and thiopronine

Cystinuria is a recessive hereditary disorder characterized by a defect in the reabsorption of dibasic amino acids (cystine, ornithine, lysine, and arginine) in the cells of the renal and intestinal tubules. Cystine is a homodimer formed by the binding of two cysteine molecules via a disulfide bond. Thiol-containing drugs (e.g. thiopronine [2-mercaptopropionylglycine], D-penicillamine) have sulfhydryl groups that can cleave this disulfide bond, resulting in the formation of cysteine mixed disulfides, which are more soluble than the cystine homodimer^115^. For patients with cystinuria who do not respond well to conservative measures, such as significant increased diuresis, vigorous alkalinization with potassium citrate, and reduced dietary sodium and methionine intake^115^, or who are likely to be resistant to them, specific treatment with thiopronine is recommended, due to its lower incidence of adverse effects compared to D-penicillamine^114,116^. However, the drawback is the unavailability of the former in Brazil. The dose of thiopronine for adults ranges from 600 to 900 mg/day (divided into 3 doses). The usual dose of Cuprimine® (D-penicillamine in 250 mg capsules) for adults ranges from 500 to 1,500 mg/day, divided into 2 to 3 doses. The daily dose should be adjusted to maintain the non-bound urine cystine concentration under 250 mg/L (1 mmol/L). If D-penicillamine is used long-term, it is important to supplement with pyridoxine (vitamin B6) at a dose of 50 mg per day, as D-penicillamine may cause a deficiency of this vitamin.

Thiol-containing drugs may cause a variety of adverse effects, including fever, rash, altered taste, arthritis, leukopenia, aplastic anemia, hepatotoxicity and pyridoxine (vitamin B6) deficiency. In addition, patients may present with proteinuria, usually associated with membranous nephropathy, rarely crescentic glomerulonephritis, or minimal change disease^116^.

#### Pyridoxine (vitamin B6) and lumasiran

Pyridoxine acts as a cofactor for the enzyme alanine-glyoxylate aminotransferase (AGT). This enzyme is responsible for converting glyoxylate into glycine in the liver. Therefore, by facilitating the conversion of glyoxylate into glycine, pyridoxine helps reduce the amount of oxalate produced in the body. Approximately up to 50% of patients with primary hyperoxaluria type 1 (PH1) will respond to pyridoxine therapy with a significant reduction in urinary oxalate excretion. This occurs most commonly in patients with homozygous mutations in AGXT p.Gly170Arg or p.Phe152Ile^164^. The dose of pyridoxine is 5–20 mg/kg divided into one or two doses daily. A positive response is defined as a reduction in urinary oxalate greater than 30%^43^.

Lumasiran is part of a novel class of drugs used to treat PH1, represented by an RNA interference (RNAi) that targets the degradation of the messenger RNA of the HAO1 gene (hydroxyacid oxidase 1), which encodes the enzyme glycolate oxidase (GO). This enzyme converts glycolate into glyoxylate and glyoxylate into oxalate. By inhibiting HAO1, lumasiran reduces the production of GO, the direct precursor of oxalate in the liver. It is administered subcutaneously, and the dosage is calculated based on body weight. A recent consensus from OxalEurope, in collaboration with the European Reference Network for Rare Kidney Diseases (ERKNet) and several other entities, outlines the options and suitability of these new therapies, as well as conventional therapy in PH1. The drug has already been approved by the FDA, the European Medicines Agency (EMA), and ANVISA based on pivotal phase 3 studies in children and adults, including even patients with more advanced stages of chronic kidney disease or even on dialysis^43^. However, the drug is not yet available for use in our country, and the high cost of the medication still compromises access to treatment for the population, as is the case with other orphan drugs.

#### Other medications

In the case of staghorn calculi, antibiotic therapy, combined with surgical removal, is the primary component for the treatment of struvite stones. Urine sterilization with antibiotic therapy may help reduce the recurrence rate of stones. In the past, acetohydroxamic acid was indicated to treat patients with residual fragments or recurrent post-surgery struvite stones, or those who could not undergo surgery^165^. However, this agent is associated with serious side effects that limit its use, which is already very rare in our context.

**Figure 5:**
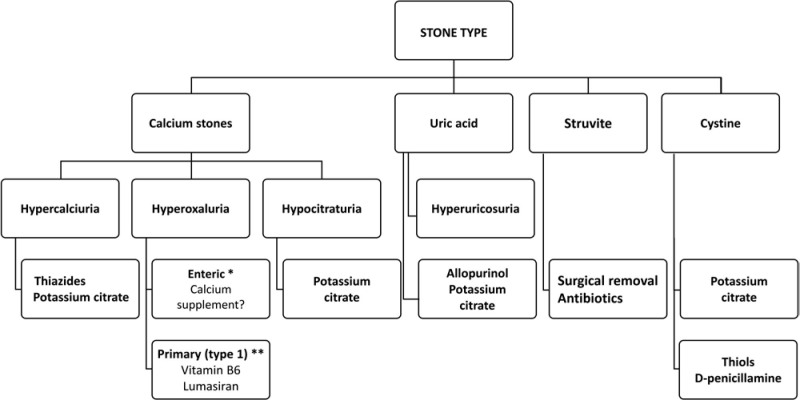
Treatment according to the stone type or metabolic disorder.

Figure 5 illustrates the treatment according to the type of stone (by analysis, imaging, or presumed) and/or metabolic disorder.

Notes – *Investigate the cause; **Genetic testing.

### Summary and Recommendations

Thiazide diuretics can be administered to patients with hypercalciuria and recurrent calcium nephrolithiasis (Grade of recommendation: B; Level of evidence: 2).

Potassium citrate is recommended for patients with hypocitraturia and recurrent calcium nephrolithiasis (Grade of recommendation: A; Level of evidence: 1).

Thiazide diuretics and/or potassium citrate can be administered to patients with recurrent calcium stones without metabolic abnormalities (Grade of recommendation: B; Level of evidence: 2).

Potassium citrate should be offered to patients with uric acid and cystine stones to increase urinary pH to an optimal level (above 6.5) (Grade of recommendation: B; Level of evidence: 2).

In patients with uric acid stones, allopurinol can be used as adjuvant therapy in patients with hyperuricemia or hyperuricosuria (Grade of recommendation: B; Level of evidence: 2).

Thiol-containing drugs can be offered to patients with cystine stones who do not respond to dietary modifications and urine alkalinization (Grade of recommendation: C; Level of evidence: 3).

In enteric hyperoxaluria, calcium supplements with food and alkaline citrate may be prescribed (Grade of recommendation: B; Level of evidence: 2).

Testing the response to pyridoxine is recommended in patients with primary hyperoxaluria type 1. In these patients, lumasiran may be indicated, preferably with the help of a specialist (Grade of recommendation: B; Level of evidence: 2).
