# Supplementary material for: Brazilian Guidelines on evaluation and clinical management of Nephrolithiasis: Brazilian Society of Nephrology
Source: J Bras Nefrol. 2025 Mar 10;47(2):e20240189. doi: 10.1590/2175-8239-JBN-2024-0189en (PMC11913452; doi:10.1590/2175-8239-JBN-2024-0189en)
Supplement: Translation

# Translation: Diretrizes Brasileiras para diagnóstico e tratamento clínico da Nefrolitíase: Sociedade Brasileira de Nefrologia

**DOI:** 10.1590/2175-8239-JBN-2024-0189pt

**Authors:** Mauricio de Carvalho, Ana Cristina Carvalho de Matos, Daniel Rinaldi dos Santos, Daniela Veit Barreto, Fellype Carvalho Barreto, Fernanda Guedes Rodrigues, Igor Gouveia Pietrobom, Lucas Gobetti da Luz, Natasha Silva Constancio, Samirah Abreu Gomes, Ita Pfeferman Heilberg

**Affiliations:** 1 Universidade Federal do Paraná, Hospital de Clínicas, Curitiba, PR, Brasil.; 2 Pontifícia Universidade Católica do Paraná, Disciplina de Nefrologia, Curitiba, PR, Brasil.; 3 Universidade Federal de São Paulo, Disciplina de Nefrologia, Escola Paulista de Medicina, São Paulo, SP, Brasil.; 4 Faculdade de Medicina do ABC, Santo André, SP, Brasil.; 5 Universidade Federal de São Paulo, Pós-Graduação em Nutrição, São Paulo, SP, Brasil.; 6 Hospital Moinhos de Vento, Departamento de Nefrologia, Porto Alegre, RS, Brasil.; 7 Hospital Unimed Vale do Sinos, Novo Hamburgo, RS, Brasil.; 8 Hospital Regional Alto Vale e Associação Renal Vida, Rio do Sul, SC, Brasil.; 9 Universidade de São Paulo, Faculdade de Medicina, São Paulo, SP, Brasil.

**Keywords:** Nefrolitíase, Cálculos Renais, Urolitíase

## Abstract

A prevalência da nefrolitíase tem aumentado nos últimos anos, afetando entre 10% e 15% da população. A doença litiásica está associada a comorbidades sistêmicas, como doença cardiovascular, diabetes *mellitus* e obesidade. A primeira Diretriz de Litíase Urinária da Sociedade Brasileira de Nefrologia foi publicada em 2002, e desde então o acúmulo de novos estudos clínicos e diretrizes justificou a revisão do tema. Este documento, elaborado pelo Comitê de Nefrolitíase da Sociedade Brasileira de Nefrologia, reflete os avanços no manejo dos pacientes com cálculos renais. A diretriz tem como objetivo fornecer recomendações para diagnóstico, prevenção e tratamento da nefrolitíase, embasadas nas melhores evidências disponíveis. Os tópicos abordados incluem avaliação clínica, exames laboratoriais e de imagem, além de intervenções dietéticas, farmacológicas e estratégias de acompanhamento.

## Introdução

A nefrolitíase é uma das condições clínico-cirúrgicas mais frequentes, caracterizada pelo surgimento de estruturas sólidas cristalinas no interior do sistema urinário. A prevalência da doença litiásica continua a aumentar globalmente, principalmente nos últimos anos, em ambos os sexos e em todas as etnias. Estima-se que afete 10% a 15% da população, com frequência maior em homens, em uma proporção de 2–3:1 em comparação com as mulheres. O pico de incidência ocorre entre a terceira e quinta décadas de vida, com taxas de recorrência, sem tratamento, de até 50% em 10 anos^1^. Uma análise de dados do National Health and Nutrition Examination Survey (NHANES) dos Estados Unidos, de 2015 a 2018, com mais de 10 mil pacientes com idade acima de 20 anos, revelou prevalência de 11% (IC 95%–10,1–12). A incidência em 12 meses foi de 2,1% (IC 95%–1,5–2,7)^2^.

Além disso, há evidências recentes de que a nefrolitíase está relacionada a outras doenças sistêmicas, principalmente doença cardiovascular, diabetes *mellitus* e obesidade. A nefrolitíase também pode estar associada ao desenvolvimento de doença renal crônica, especialmente em indivíduos do sexo feminino e em pacientes com sobrepeso^3^.

O aumento progressivo na prevalência e os custos associados à nefrolitíase a tornaram uma das condições mais comuns e impactantes encontradas atualmente na prática médica. A prevenção continua sendo a melhor maneira de evitar os cálculos urinários, especialmente dada a sua alta taxa de recorrência.

A avaliação do indivíduo litiásico requer uma abordagem clínica abrangente, visando identificar fatores ambientais, metabólicos e/ou genéticos relaci­onados à formação dos cálculos. Além disso, são necessários estudos de imagem para avaliar e monitorar a presença de cálculos no trato urinário, bem como análises laboratoriais (composição bioquímica do sangue e urina, análise da composição dos cálculos) para direcionar as estratégias terapêuticas, tanto nutricionais quanto farmacológicas.

É de se lamentar que nenhuma das sociedades mais influentes de nefrologia (ASN, ISN, ERA) lançou diretrizes específicas sobre urolitíase até este momento^4^. Em 2002, foi publicada a primeira Diretriz de Litíase Urinária da Sociedade Brasileira de Nefrologia^5^. Neste longo período, atualizações de várias diretrizes sobre o tema, juntamente com a publicação de inúmeros estudos clínicos, geraram a necessidade de revisão do tema. Este trabalho foi realizado pelo Comitê de Nefrolitíase da Sociedade Brasileira de Nefrologia e visa refletir os avanços no manejo de pacientes com cálculos renais. Deve ser enfatizado que este documento reflete as melhores evidências selecionadas e disponíveis para os membros desse comitê. Porém, as diretrizes não substituem a expertise clínica na tomada de decisões para pacientes individuais, mas ajudam a direcionar essas decisões.

Embora não seja explicitamente sugerido, a publicação desta diretriz constitui um apelo para a cogestão do nefrologista no cuidado longitudinal e holístico de pacientes com urolitíase. E, idealmente, em cooperação com outros profissionais de saúde, como médicos de atenção primária, internistas, nutricionistas, urologistas, entre outros^4^.

## Métodos

As informações presentes neste documento fo­ram obtidas por meio de revisão da literatura, principalmente em língua inglesa. Para esta Diretriz, evidências foram identificadas, compiladas e analisadas por uma avaliação estruturada dos artigos.

As recomendações foram baseadas, sempre que possível, na literatura mais recente desde a publicação da última diretriz, em 2002^5^. O intervalo de pesquisa foi estendido até junho de 2024, incluindo alguns dos seguintes termos no título ou resumo: “nephrolithiasis“, “urolithiasis”, “kidney stone”, “renal stone”, “urinary stone”, “urinary calculi”.

A busca foi orientada prioritariamente, embora não de maneira exclusiva, para estudos que representassem níveis elevados de evidência, como revisões sistemáticas com metanálise, ensaios clínicos randomizados e estudos comparativos prospectivos não randomizados. Além disso, foram analisadas referências de diretrizes recentemente publicadas. Foram realizadas revisões separadas da literatura para cada um dos principais tópicos^6^.

Foi utilizado o sistema de classificação de Níveis de Evidência do Centro de Medicina Baseada em Evidências da Universidade de Oxford, de 2011, para avaliar o nível de evidência das recomendações incluídas no documento^7^, conforme mostrado na Tabela 1. As recomendações foram baseadas na revisão especializada da literatura e representam o consenso dos autores desta diretriz. Todos os especialistas envolvidos apresentaram declarações de potenciais conflitos de interesse.

**Tabela 1:** Sistema de classificação – níveis de evidência

Grau de recomendação	Descrição
A	Estudos randomizados controlados de alta qualidade.
B	Estudos observacionais bem conduzidos e ensaios clínicos randomizados de baixa qualidade.
C	Estudos de caso-controle e séries de casos.
D	Opinião de especialistas.
Nível de evidência	Descrição
1	Evidências derivadas de meta-análises de ensaios clínicos randomizados ou pelo menos um ensaio clínico randomizado bem conduzido.
2	Evidências derivadas de pelo menos um ensaio clínico bem conduzido ou estudos observacionais de alta qualidade com consistência entre eles.
3	Evidências derivadas de estudos observacionais, séries de casos ou relatos de casos.
4	Evidências derivadas de opiniões de especialistas baseadas em experiência clínica, estudos descritivos ou relatórios de comitês de especialistas.

## Cólica Reno-Ureteral Aguda

### Quadro Clínico

A nefrolitíase pode se manifestar de forma totalmente assintomática, sendo diagnosticada incidentalmente através de exames de imagem, ou pode causar apenas uma dor vaga nos flancos. No entanto, a apresentação característica é a cólica ureteral, que geralmente começa com uma dor súbita, intensa e unilateral. Essa dor aumenta e diminui em intensidade, desenvolvendo-se em ondas ou paroxismos que tipicamente duram de 20 a 60 minutos^8^. A gravidade da dor está mais relacionada à velocidade de instalação da obstrução do que à sua intensidade. Essa dor é comumente localizada na região lombar, no flanco ou na fossa ilíaca. Não é aliviada pelo repouso ou mudança de posição e pode irradiar para o trajeto ureteral, região da bexiga e genitália externa. Outros sintomas podem incluir disuria e hematúria macroscópica. Sintomas gastrointestinais também podem ocorrer de forma variável, como náuseas (44%), vômitos (51%), anorexia (54%), constipação ou diarreia (10%)^9^.

Outras formas de apresentação clínica da nefrolitíase incluem a hematúria micro ou macroscópica, não glomerular (isomórfica). No entanto, a hematúria pode não ser detectada em aproximadamente 10% a 30% dos pacientes com cólica nefrética^10^. O intervalo desde o início da dor aguda até o momento do exame de urina é um fator que pode prejudicar a sensibilidade da hematúria. Em um estudo retrospectivo de mais de 450 pacientes com ureterolitíase aguda documentada por tomografia computadorizada (TC), a hematúria estava presente em 95% no dia 1, e 65% a 68% nos dias 3 e 4^11^.

A nefrolitíase pode se apresentar também como a eliminação espontânea de cálculos sem a presença de dor, ou por meio de infecções urinárias recorrentes, especialmente aquelas causadas por bactérias do gênero *Proteus*. A combinação de dor lombar, febre, calafrios e sepse pode ocorrer na pielonefrite obstrutiva calculosa, uma condição com elevada morbidade e mortalidade.

Ao exame físico, podem ser observadas taquicardia, palidez, sudorese, dor à palpação em região de ângulo costovertebral (sinal de Giordano, sensibilidade de 15%, especificidade de 99% e razão de verossimilhança de +30) e distensão abdominal leve, porém sem sinais de irritação peritoneal^12^. O quadro clínico é sugestivo, mas é importante realizar o diagnóstico diferencial com patologias gastrintestinais, ginecológicas, urológicas, afecções vasculares e outras causas médicas de abdome agudo.

O *STONE score* é uma ferramenta de predição clínica que ajuda a identificar pacientes com quadro de dor abdominal aguda com probabilidade muito baixa ou muito alta de cálculo ureteral não complicado. Desse modo, auxilia na decisão de complementação de investigação para diagnósticos alternativos e solicitação de exame de imagem. As variáveis analisadas no protocolo são: sexo, raça, duração da dor, náuseas e/ou vômitos e presença de hematúria^13^.

### Resumo e Recomendações

A associação de dor abdominal de início recente (até 12 horas), sensibilidade em região lombar ou ureteral e presença de hematúria é sugestiva de cólica renal aguda. Portanto, a obtenção de história clínica detalhada e exame físico cuidadoso são de extrema importância (Grau de recomendação: B; Nível de evidência: 2).

Escores diagnósticos, como o STONE, podem ser utilizados como auxílio na tomada de decisão quando houver incerteza no diagnóstico e necessidade de tratamento imediato (Grau de recomendação: B; Nível de evidência: 2).

### Investigação Complementar

Recomenda-se a confirmação da presença de cálculo urinário e sua localização. A avaliação imediata por imagem é indicada em pacientes com febre, portadores de rim único e quando o diagnóstico é duvidoso. No entanto, o alívio da dor ou qualquer outra medida de emergência não devem ser adiados para a realização de exames de imagem^14^.

A radiografia simples de abdome (Rx) é frequente­mente utilizada porque cerca de 90% dos cálculos renais têm densidade radiopaca. Para ser visível, é necessário que o cálculo tenha pelo menos 2 mm em seu maior diâmetro. No entanto, as radiografias simples têm limitações de sensibilidade para detectar cálculos ureterais. Alguns estudos relatam sensibilidade inferior a 50%. Apesar disso, ainda são consideradas ferramentas úteis para monitorar a progressão de cálculos radiopacos, especialmente quando interpretadas em conjunto com a ultrassonografia^15,16^.

A ultrassonografia pode detectar todos os tipos de cálculos renais, independentemente da radiopacidade, além de avaliar a presença e o grau de eventual hidronefrose. Essa modalidade de exame pode ser realizada tanto durante episódios de cólica nefrética quanto durante a gestação. A ultrassonografia apresenta sensibilidade reduzida para detecção de cálculos ureterais (sensibilidade de 45% e especificidade de 94%) e está sujeita à dependência do equipamento utilizado e da habilidade do operador em interpretar as imagens^17^.

A tomografia computadorizada (TC) é o padrão-ouro para o diagnóstico de litíase urinária. Uma metanálise revelou que TC de baixa dose diagnosticou urolitíase com sensibilidade de 93,1% (IC 95%: 91,5–94,4) e especificidade de 96,6% (IC 95%: 95,1–97,7%). Além disso, permite avaliar o abdome em poucos minutos, sem necessidade de contraste, pode diagnosticar doenças não relacionadas ao trato urinário e visualizar quase todos os tipos de cálculo (radiopacos ou não)^18^. Também possibilita utilizar a densidade, medida em unidades Hounsfield, para estimar a composição do cálculo (em escala crescente de densidade – ácido úrico, estruvita, cistina, oxalato de cálcio monoidratado e hidroxiapatita) e sua resposta ao tratamento (cálculos com densidade maior que 1.000 unidades Hounsfield apresentam difícil fragmentação à litotripsia extracorpórea)^19^. Suas desvantagens são a maior exposição à radiação e o maior custo^20^. A ressonância magnética tem aplicação limitada na investigação da calculose urinária, sendo reservada somente para situações especiais em grávidas e crianças^21^. O hemograma normalmente mantém-se inalterado ou pode apresentar leucocitose discreta durante a fase aguda, sem desvio significativo para a esquerda. No entanto, em pacientes mais idosos com aumento acentuado nos níveis de proteína C reativa (PCR), a suspeita de infecção deve ser considerada, e o tratamento com antibióticos pode ser necessário^22^. A concentração sérica de creatinina é geralmente normal, exceto em situações como cálculo obstrutivo em rim único, obstrução bilateral do ureter, presença de cálculos vesicais de grande tamanho ou cálculos uretrais impactados. Leucócitos podem ser encontrados na urina tipo I, porém a presença de nitrito positivo e de bactérias no exame do sedimento deve levantar a suspeita de infecção associada. Hematúria micro ou macroscópica é comum na cólica nefrética, mas sua ausência não exclui o diagnóstico.

A Figura 1 resume as etapas para investigação de suspeita de cólica renal.

**Figura 1:** Investigação de suspeita de cólica renal.

Notas – *Diagnósticos alternativos com relevância clínica: Ruptura ou dissecção de aneurisma de aorta, malignidade (renal, ovariana), diverticulite, apendicite, colecistite, obstrução intestinal, torção ovariana/testicular, gestação ectópica, trombose/infarto renal, hematoma retroperitoneal, embolia pulmonar, pneumonia. **Sinais de alerta: Primeiro episódio >50 anos, febre, alterações hemodinâmicas, rim único, disfunção renal, quadro atípico de cólica renal.

### Resumo e Recomendações

A realização imediata de exames de imagem é indicada quando há dúvida no diagnóstico diferencial com outras causas de lombalgia ou quando em ausência de hematúria, em casos de febre ou presença de rim único reportado pelo paciente. A ultrassonografia, em conjunto com a radiografia simples do abdome, pode ser considerada a modalidade inicial para a cólica nefrética (Grau de recomendação: A; Nível de evidência: 1).

A tomografia computadorizada, preferencialmente em protocolo de baixa dose, fornece informações valiosas para confirmação diagnóstica e decisão terapêutica, com vantagens nos casos de identificação de cálculos em região ureteral, bem como de determinar a repercussão do cálculo sugerindo ou não obstrução. Ressalta-se que a ultrassonografia também pode indicar presença de dilatação como repercussão da presença do(s) cálculo(s) (Grau de recomendação: A; Nível de evidência: 1).

No atendimento de emergência, recomenda-se avaliação bioquímica sucinta, com dosagem de crea­tinina, hemograma e urina tipo I. Idade do paciente (>54 anos) e níveis elevados de PCR (>5 vezes o valor de referência) ajudam a decidir a prescrição de antibióticos, na suspeita de infecção associada à cólica nefrética (Grau de recomendação: C; Nível de evidência: 4).

### Tratamento

Acredita-se que a dor da cólica renal seja causada pelo espasmo da musculatura lisa do ureter, associado a edema e inflamação decorrentes da obstrução causada pelo cálculo. Além disso, o aumento da peristalse e da pressão proximal ao cálculo contribuem para a intensificação da dor^23^. As duas principais classes de medicações utilizadas para analgesia na cólica renal são os anti-inflamatórios não hormonais (AINH) e os opioides. Os AINH, ao inibirem a síntese de prostaglandinas, reduzem a inflamação e a hiperatividade muscular ureteral. Em um estudo publicado em 2016, 1500 pacientes adultos com cólica nefrética foram randomizados para receber diclofenaco intramuscular, morfina intravenosa ou paracetamol intravenoso. Após 30 minutos, os AINH foram 50% mais eficazes na redução da dor em comparação com a morfina, sem eventos adversos. Uma limitação do estudo foi a dose de morfina (0,1 mg/kg/IV), abaixo da normalmente utilizada^24^. Em meta-análise recente, demonstrou-se que os pacientes tratados com AINH obtiveram redução nos escores de dor semelhante àqueles tratados com opioides, porém com menos efeitos colaterais^25^. O cetoprofeno é um dos AINH comumente utilizados, com boa ação analgésica e que pode ser administrado via intravenosa. Outros AINH, como diclofenaco, ibuprofeno ou indometacina, também apresentam bom nível de evidência para sua utilização^26^.

Deve-se lembrar, entretanto, que os AINH apresentam contraindicação absoluta ou relativa em situações como insuficiência renal, doença péptica grave e gestação – nesses casos, deve-se considerar os opioides. A morfina é o representante clássico dessa classe de medicamentos e, apesar de não atuar na fisiopatogenia da cólica ureteral, apresenta ação analgésica rápida, potente e titulável. Porém, tem efeitos colaterais, como náuseas, obstipação intestinal, entre outros, além de retenção urinária, depressão respiratória e, em doses mais elevadas^27^, hipotensão. O cloridrato de tramadol causa menos sedação, porém com menor efeito analgésico. O cloridrato de meperidina induz vômitos frequentes e sua ação pode se prolongar ou ser potencializada na insuficiência renal^28^. Os antiespasmódicos, como a hioscina, apresentam efeito controverso e limitado, mesmo quando utilizados em associações a outros analgésicos^29^.

Apesar de os Estados Unidos e a Inglaterra terem banido o uso da dipirona em função do raro aparecimento de agranulocitose, esse agente é a droga não opioide mais popularmente usada para analgesia em outros países incluindo o Brasil, seja em associação com a hioscina ou isoladamente, com bons resultados, apesar dos efeitos colaterais relacionados à sonolência.

Embora seja útil reidratar pacientes hipovolêmicos, com náuseas e vômitos significativos, ou naqueles com suspeita de lesão renal aguda pré-renal, a hidratação intravenosa com o único propósito de promover a passagem forçada de cálculos não é respaldada pela literatura e deve ser evitada^30^.

A eliminação espontânea de cálculos ureterais ocorre em até 80% dos cálculos menores que 5 mm. Para cálculos maiores que 7 mm, a chance é bem menor, em torno de 25% para os localizados em ureter proximal, 45% para aqueles em ureter médio e 70% para cálculos de ureter distal^31^.

Os bloqueadores de receptores alfa-1-adrenérgicos (ex., tansulosina) têm sido utilizados como facili­tadores da passagem espontânea de cálculos ureterais, principalmente aqueles localizados no ureter distal e menores que 1 cm. Vários estudos da chamada terapia expulsiva médica (TEM), a maioria randomizados e controlados, porém com pequeno número de pacientes, demonstraram que os alfa-bloqueadores aceleram a passagem do cálculo, diminuem a dor e, consequentemente, reduzem a necessidade de analgésicos, com mínimos efeitos colaterais (hipo­tensão, principalmente na primeira dose)^32^. Porém, um estudo multicêntrico, que avaliou mais de 1.000 pacientes, revelou que a TEM se assemelhou ao placebo no número de intervenções necessárias para eliminação de cálculos ureterais. Nesse estudo, 75% dos cálculos tinham diâmetro < 5mm e a maioria (65%) estava localizada no ureter inferior^33^. Mais recentemente, uma nova metanálise apenas de estudos randomizados e controlados demonstrou chance 44% maior de eliminação de cálculos ureterais distais de 5–10 mm com a utilização de tansulosina^34^. A tansulosina, assim como o citrato de potássio (em dose média de 55 mEq/dia), também é utilizada em alguns protocolos como terapêutica complementar após litotripsia extracorpórea para acelerar a eliminação de fragmentos de cálculo^35^.

A Figura 2 ilustra as etapas no manejo da cólica renal e a Tabela 2 resume as principais medicações utilizadas na cólica renal.

**Figura 2:** Manejo da cólica renal.

**Tabela 2:** Principais medicações utilizadas no tratamento da cólica renal no pronto socorro

Classe	Apresentação (dose adultos)	Observações (efeitos colaterais)	Contraindicações
***Analgésicos***			
Paracetamol	**Comp. 500–750 mg:** 1 comp. VO 4-6x/dia (Dmáx: 4000 mg/dia)	Hepatotoxicidade, alteração de transaminases	*Absolutas:* hipersensibilidade *Relativas:* deficiência de G6PD, doença hepática em atividade
Dipirona	**Comp. 500–1000 mg:** (2 comp. VO 4x/dia) **Ampola 500 mg/mL (2 mL):** 1-2 ampolas EV 4x/dia	Anafilaxia, hipotensão arterial, agranulocitose	*Absolutas:* hipersensibilidade *Relativas:* gestantes e lactantes, doença medular, deficiência de G6PD
***Antiespasmódicos***			
Escopolamina	Drágeas 10 mg: 1 a 2 drágeas 4x/dia (Dmáx VO: 60 mg/dia) Ampola 30 mg/mL (1 mL): 1 a 2 ampolas EV, IM ou SC 4-6x/dia (Dmáx EV: 100 mg/dia)	Xerostomia, borramento visual, retenção urinária, sonolência, fadiga, alterações psiquiátricas (agitação, confusão mental, alucinações). As complicações costumam ocorrer em idosos e em doses mais elevadas.	*Absolutas:* hipersensibilidade, glaucoma, megacólon, íleo paralítico, hiperplasia prostática com retenção urinária, lesões estenóticas gastrointestinais, miastenia gravis, taquicardia por ICC
***Anti-inflamatórios não esteroidais***			
Cetorolaco	**Comp. 10 mg:** 20 mg SL de ataque, 10 mg 4-6x/dia (Dmáx VO: 40 mg/dia) **Ampola 30 mg/mL:** 30 mg EV ou 360 mg IM (Dmáx EV: 120 mg/dia)	Dispneia, náuseas, dor abdominal, diarreia, cefaleia, tontura, alteração transaminases, sonolência, zumbido. Anafilaxia, hemorragia gastrointestinal, disfunção renal, broncoespasmos, farmacodermia, agranulocitose	*Absolutas:* hipersensibilidade, úlcera péptica ativa, hemorragia cerebrovascular, 3º trimestre de gestação ou lactante *Relativas:* idosos, hipertensão, ICC
Cetoprofeno	**Cáps. 500–1000mg:** 50 mg VO 6/6 hr ou 100 mg VO 12/12 hr **Ampola 100 mg:** 100 mg EV (diluir em 100 mL de SF/SG por infusão EV em 20 min)		*Absolutas:* hipersensibilidade, úlcera péptica ativa, hemorragia gastrointestinal, 3º trimestre de gestação ou lactante *Relativas:* idosos, falência renal ou hepática, ICC
***Opioides***			
Tramadol	**Comp. 50–100 mg:** VO **Ampola 50 mg/mL (2 mL):** 50 a 100 mg EV ou IM 6/6 hr (Dmáx 400 mg/dia) (diluir 1 mg/mL SF/SG em infusão lenta em 30 a 60 min)	Tontura, sonolência, sedação, cefaleia, agitação, diaforese, constipação, dispepsia, náuseas, vômitos, xerostomia, retenção urinária, bradicardia, hiponatremia, crise convulsiva, bradicardia, miose, depressão respiratória (menor risco com uso de Tramadol comparado a outros opioides)	*Absolutas:* hipersensibilidade, obstrução gastrointestinal incluindo íleo paralítico, uso de IMAO nos 14 dias antecedentes *Relativas:* idosos, depressão respiratória, alcoolismo. Usar com cautela em portadores de insuficiência hepática ou renal
Meperidina	**Ampola 50 mg/mL (2 mL):** 1 mg/kg IM (preferencialmente) 4/4 hr (Dmáx 500 mg/dia) Dose de ataque de 25–50 mg EV (diluir em 10 mL SF/SG lento ou SC (se necessário)		
Morfina	**Comp. 10 mg:** 5 a 30 mg VO 4/4 hr **Ampola 10 mg/mL:** (1 mL) **Ampola 1 mg/mL (2 mL):** 1 mg/kg IM ou EV 4/4 hr		
***Bloqueador alfa***			
Tansulosina	**Comp.0,4 mg:** VO 1x/dia	Tontura, hipotensão ortostática, distúrbios na ejaculação, cefaleia, rinite	*Absolutas:* hipersensibilidade, uso concomitante com forte inibidor CYP3A4 (por ex.: cetoconazol)

### Resumo e Recomendações

Os AINH são eficazes no tratamento da cólica renal e superiores aos opioides. O uso de opioides para o manejo de cólica renal deve ser minimizado (Grau de recomendação: A; Nível de evidência: 1).

A hidratação intravenosa com o único propósito de promover a passagem forçada de cálculos deve ser evitada (Grau de recomendação: B; Nível de evidência: 1).

O papel da terapia médica expulsiva (TEM) na promoção da passagem espontânea de cálculos renais é controverso, mas a literatura atual sugere benefício em cálculos ureterais distais, com tamanho entre 5 e 10 mm. As vantagens e desvantagens da TEM devem ser discutidas com o paciente em um processo de tomada de decisão compartilhada (Grau de recomendação: A; Nível de evidência: 1).

## Investigação Metabólica

### Quem Investigar?

O objetivo do diagnóstico laboratorial de pacientes formadores de cálculos com base na atividade metabólica é identificar de maneira eficiente e custo-efetiva diferenças comportamentais e fisiopatológicas distintas para um planejamento terapêutico e prevenção da recorrência de litíase mais eficazes. O tipo e a extensão da avaliação vão depender da gravidade do quadro; da distinção entre formadores únicos, recorrentes ou ocasionalmente recorrentes; das evidências de doença sistêmica e/ou fatores de risco associados à recorrência; da presença de história familiar de nefrolitíase (NL); do interesse do paciente na prevenção de cálculos, e finalmente da entidade financiadora (SUS, convênio ou recursos próprios).

Por definição, formadores de cálculos únicos são os pacientes com episódio de único e solitário cálculo, sintomático ou não. Nefrolitíase recorrente é definida por 2 ou mais episódios de cálculos, sincrônicos ou em diferentes momentos temporais. Os cálculos ocasionalmente recorrentes envolvem a categoria de pacientes que recorrem com intervalo superior a 5 anos e que poderiam se apresentar como uma categoria de risco intermediária entre formadores únicos e recorrentes. Entretanto, como as anormalidades metabólicas são similares nos formadores de cálculos únicos ou recorrentes, a complexidade do estudo metabólico deverá ser regida pelas características clínicas elencadas acima ou pelo custo-efetividade^36,37^. Rule et al.^38^ estimaram taxas de recorrência após 2, 5,10 e 15 anos de 11%, 20%, 31% e 39%, respectivamente, e identificaram como fatores de risco a idade jovem, sexo masculino, etnia branca, história familiar, cálculos prévios assintomáticos ou não obstrutivos, cálculo renal pélvico sintomático ou de polo inferior em exames de imagem, hematúria macroscópica e composição do cálculo de ácido úrico. Tais dados podem ser utilizados em um nomograma (nomograma de ROKS) para estimativa do risco de recorrência e posterior tomada de decisão compartilhada diagnóstica e terapêutica^38^. Entretanto, tal abordagem não foi validada em outras populações e, em especial, em brasileiros.

Outra distinção importante é entre o formador de cálculo *metabolicamente ativo* (nova formação de cálculo ou crescimento de cálculo em imagens seriadas, e/ou eventos clínicos relacionados à nefrolitíase durante o seguimento) e o formador de cálculo *metabolicamente inativo* (sem atividade aparente de cálculo em exames de imagem e no seguimento clínico e de acompanhamento)^37^.

Em acordo com diretrizes internacionais, recomenda-se que formadores de cálculos recorrentes e formadores de cálculo único de alto risco (aqueles com fatores de risco listados acima) ou formadores de cálculos pela primeira vez interessados em prevenção de eventos futuros devam ser direcionados a uma avaliação metabólica individualizada^39,40^.

A avaliação laboratorial de pacientes com NL baseia-se em exames séricos gerais, envolvendo os bioquímicos e hormonais, visando também à investigação de distúrbios eletrolíticos e ácido-básicos, exames de urina simples ou sedimento urinário (sinonímias: Urina tipo 1, EAS, EQU) e avaliação metabólica em urinas de 24h^37^. As avaliações laboratoriais são usadas para orientar a escolha do tratamento inicial, identificando também a existência de outros diagnósticos de tubulopatias e endocrinopatias associadas à NL e/ou a nefrocalcinose (NC).

### Como Investigar?

#### Bioquímica sérica

Conforme apresentado na Tabela 3, seguem os principais exames a serem solicitados. Os valores de referência representam apenas uma sugestão, pois variam com a padronização de cada laboratório e também diferem entre as populações, dificultando as comparações com as diretrizes internacionais. A seguir, o racional das solicitações:

**Tabela 3:** Parâmetros laboratoriais séricos em adultos

Parâmetros séricos	Valor de referência	Relevância no diagnóstico
Creatinina	0,6–1,0 mg/dL (F) 0,8–1,2 mg/dL (M)	Cálculo da TFGe (taxa de filtração glomerular estimada)
Sódio	135–145 mEq/L	Descartar hiponatremia
Potássio	3,0 –5,0 mEq/L	Influência no tratamento - Hipocalemia (checar ATR, tubulopatias)
Cálcio	8,6–10,2 mg/dL (cálcio total)	**Elevado:** HPTP (sempre avaliar simultâneo com PTH)
	1,12–1,32 mmol/L (cálcio ionizado)	**Reduzido:** Hipoparatireoidismo
Ácido úrico	4,6 mg/dL	**Elevado:** Gota, síndrome metabólica **Reduzido:** Xantinúria
Cloro	98–112 mmol/L	Cálculo do ânion Gap
Fósforo	3,0–4,5 mg/dL	**Reduzido:** tubulopatias perdedoras de fosfato (calcular TmP)/HPTP **Elevado:** DRC ou Hipoparatireoidismo
Magnésio	1,6–2,6 mg/dL	Tubulopatias (Bartter, FHHNC)
Oxalato	<20,0–26,6 mol/L	Casos de NC ou NL recorrente com TFGe < 30 mL/min/1,73m^2^
Gasometria venosa	pH: 7,35–7,45 pO_2_: 30–50 mmHg pCO_2_: 35–45 mmHg HCO_3_: 22–26 mmol/L BE: +2 mmol/L	Valorizar HCO3 para estados de acidose (ATR, etc.) ou alcalose (Bartter, etc.)
PTH	20–66 pg/mL (40–49 anos) 21–71 pg/mL (50–59 anos) 22–84 pg/mL (>59 anos)	**Elevado:** HPTP (sempre avaliar simultâneo com cálcio) **Reduzido:** hipoparatireoidismo
25(OH)D_3_	>20 ng/mL (população sadia) >30 ng/mL (risco para perda óssea)	Hipovitaminose D Redução de densidade óssea/*1,25(OH)2D*_3_ *) em doenças genéticas*

Abreviações: ATR: acidose tubular renal; HPTP: hiperparatireoidismo primário; PTH: paratormônio; TmP: transporte tubular máximo de fósforo.

#### Creatinina

A medida de creatinina sérica é fundamental para avaliação da função renal em pacientes litiásicos, por meio do cálculo da Taxa de Filtração Glomerular estimada (TFGe) pelo CKD-EPI 2021. A Doença Renal Crônica (DRC) pode causar alterações eletrolíticas secundárias, dificultando o diagnóstico das alterações metabólicas envolvidas na nefrolitíase ou das tubulopatias primárias relacionadas à litíase renal. Por outro lado, a NL também pode evoluir para DRC. Os principais fatores de risco relacionados são malformações do trato urinário, cálculos compostos por cistina, xantina ou estruvita, infecções frequentes do trato urinário, sobrepeso, sexo feminino, rim único, nefropatia obstrutiva, doenças genéticas como a hiperoxalúria primária, acidose tubular renal ou outras tubulopatias hereditárias com nefrocalcinose.

#### Sódio (Na)

A determinação do Na sérico é importante para avaliação das tubulopatias e para o cálculo do ânion gap, utilizado por sua vez no diagnóstico das acidoses. Além disso, um estudo populacional sugeriu que hiponatremia recente e persistente também possa se associar com maior ocorrência de litíase renal^41^. Em contextos agudos, a hipernatremia pode indicar contração volêmica às custas de perda de água livre.

#### Potássio (K)

Assim como o sódio, a determinação do K sérico é importante para avaliação das tubulopatias e distúrbios ácido-básicos associados à NL já que a hipocalemia é uma manifestação comum em doenças monogênicas (e.g. Síndrome de Bartter ou acidose tubular renal distal etc.).

#### Cálcio (Ca)

A elevação do Ca sérico pode resultar em hipercalciúria, que se associa a um maior risco de formação de cálculos renais. A causa mais comum de hipercalcemia é o hiperparatireoidismo primário, caracterizado pela presença concomitante de cálcio sérico e paratormônio (PTH) elevados. Doenças monogênicas relacionadas ao metabolismo da 25(OH)-hidroxi-vitamina D, 25(OH)D ou da 1,25-dihidroxivitamina D, 1,25(OH)D, como a deficiência da hidroxilase CYP24A1, podem cursar com hipercalcemia. Por outro lado, no hipoparatireoidismo primário ou secundário (pós-cirurgia de paratireoide) e em outras desordens monogênicas, pode ocorrer hipocalcemia severa e sintomática, acompanhada de hipercalciúria, resultando em NL ou NC. Alterações confirmadas no Ca total (ajustado para a albumina) ou na dosagem de cálcio iônico tornam imperativa a avaliação de todo eixo do metabolismo do cálcio para investigação do acima exposto.

#### Ácido úrico

A hiperuricemia é comum em pacientes com síndrome metabólica ou diabetes *mellitus*, que por sua vez se associa a um pH urinário reduzido (ácido), resultando em litíase úrica. O diagnóstico de gota deve ser lembrado, na dependência de história clínica sugestiva, principalmente em pacientes com cálculos radiotransparentes ou com composição de ácido úrico em sua análise física/cristalográfica. Por outro lado, níveis muito baixos de ácido úrico podem sugerir doenças monogênicas associadas à NL, como a xantinuria.

#### Cloro (Cl)

A dosagem de Cloro, assim como o Na e o bicarbonato (HCO3), é útil para o cálculo do ânion gap na avaliação dos distúrbios ácido-básicos ligados à NL/NC. Entretanto não figura entre as dosagens de primeira linha e extrema necessidade na NL.

#### Fósforo (P)

A hipofosfatemia como fator de risco para a litíase renal em adultos é mais comum no contexto do hiperparatireoidismo primário, mas também denota possíveis distúrbios genéticos monogênicos associados à fosfatúria, raquitismo e nefrocalcinose. Na suspeita de tubulopatias monogênicas, a dosagem do P sérico é extremamente importante, em conjunto com seu nível medido em urina de amostra isolada (e com a determinação de creatinina na mesma amostra), para o cálculo da fração de excreção de fósforo e do transporte tubular máximo de P.

#### Bicarbonato (Hco3)

A mensuração do bicarbonato, realizada idealmente por gasometria venosa, é fundamental no diagnóstico de distúrbios ácido-básicos relacionados tanto à alcalose (e.g. síndrome de Bartter e Gitelman) quanto à acidose, por exemplo na acidose tubular renal (ATR) distal, associados à NL ou NC, podendo auxiliar também no diagnóstico suplementar das tubulopatias complexas. Porém, ressalta-se que, no caso da ATR distal, a gasometria deve ser realizada no mesmo dia da medida do pH urinário de 12 horas de jejum (segunda micção).

#### 25(OH)-vitamina D (25(OH)D)

A dosagem da 25(OH)D, além de avaliar o status de vitamina D, quando em conjunto com o Ca, P e PTH, é parte importante na avaliação do metabolismo ósseo, fundamental no contexto da nefrolitíase cálcica. Alterações em seus níveis podem também auxiliar no contexto de doenças monogênicas que envolvem o metabolismo da vitamina D em associação com a NL e por vezes complementadas pelas dosagens do seu metabólito ativo, a 1,25(OH)_2_D_3_.

#### Paratormônio (PTH)

A associação de NL com hiperparatireoidismo primário (HPTP) deve ser sempre buscada quando há elevação do cálcio sérico (vide acima), mas também pode se elevar secundariamente no contexto de redução da função renal e hipovitaminose D, que devem ser afastadas. O HPTP é uma das causas mais comuns de nefrocalcinose em adultos. Os valores de referência atuais variam com a idade^42^, conforme mostrado na Tabela 3.

#### Magnésio (Mg)

A hipomagnesemia pode se associar a tubulopatias genéticas associadas à nefrolitíase, como na síndrome de Bartter, mas também pode sugerir outras doenças monogênicas, como a hipomagnesemia familiar com hipercalciúria e nefrocalcinose (FHHNC), relacionada a quadros de NC importante.

#### Oxalato

O oxalato sérico deve ser dosado em todo paciente com NL recorrente ou NC, de início em idade precoce e histórico familiar, para afastar hiperoxalúria primária (HP1), quando a TFGe estiver abaixo de 30mL/min/1,73m^2^, pois, nessa condição, o oxalato urinário pode não estar elevado^43^.

#### Bioquímica urinária de 24 horas e por amostra isolada

A avaliação em urina de 24 horas para um perfil meta­bólico basal deve ser obtida pelo menos 3 a 8 semanas após a última eliminação de cálculo, espontânea ou cirúrgica (desde que o paciente esteja sem catéter duplo J). Os pacientes submetidos à avaliação metabólica devem apresentar o trato urinário desobstruído, em sua dieta habitual e sem infecção do trato urinário em curso terapêutico atual, comprovada por urocultura. Recomenda-se, de acordo com as diretrizes mais recentes, que ao menos duas amostras de urina de 24 horas sejam consideradas para o diagnóstico, sob dieta habitual, coletadas em 2 dias não consecutivos para uma avaliação metabólica em formadores de cálculos recorrentes, formadores de cálculos únicos com alto risco de recorrência, portadores de rim único ou em casos de NC^44,45,46^. Todos os analitos adiante listados podem e devem (pelas comparações entre os analitos – por exemplo Na e Ca) ser dosados numa mesma amostra, sem a necessidade de conservantes (ácidos ou alcalinos) nos recipientes, reduzindo a inconveniência e o custo de mais de duas coletas de urina de 24 horas por paciente^44,47^, e interferindo sobre a dosagem de pH em urina de 24 horas^47^. Os conservantes podem ser adicionados pós-coleta, já no laboratório, sem prejuízo dos resultados^48,49^. Para evitar a contaminação de urina e sua interferência sobre a dosagem de citrato, timol ou ácido bórico podem ser posteriormente adicionados^44,49^. Dentro de 3 a 6 meses após o início do tratamento, uma nova coleta de urina de 24 horas pode ser requisitada, para avaliar resposta à terapia dietética e/ou farmacológica e monitorar eventos adversos^37, 39^. Posteriormente, o acompanhamento pode ser anual, para avaliar a eficácia e adesão da terapia. Entretanto, de acordo com Fink et al.^39^, não há evidência científica na forma de estudos clínicos de que a normalização dos testes bioquímicos de acompanhamento são substitu­tos válidos para prever diminuição na recorrência de cálculos. Portanto, seu acompanhamento deve ser individualizado de acordo com possíveis etiologias subjacentes. Os fatores de risco considerados são início precoce de NL, histórico familiar, recorrência, cálculo de brushita (fosfato de cálcio), ácido úrico ou relacionados à infecção urinária (estruvita)^46^. Além desses, as anormalidades anatômicas do trato urinário, doenças sistêmicas ou genéticas e fatores ambientais também contribuem para elevar o risco de recorrência. Os principais analitos a serem avaliados em urina de 24 horas são mostrados na Tabela 4. Os valores de referência representam apenas uma sugestão, pois variam com a padronização de cada laboratório e também diferem entre as populações, dificultando as comparações com as diretrizes internacionais. Foram assinalados os níveis de atenção e de ação para valores de cortes (*cutoff*) distintos dos vários parâmetros. Obviamente, deve-se considerar o risco de recorrência da NL individualmente para cada paciente. Decisões terapêuticas podem ser empregadas a partir do nível de atenção e são recomendadas a partir do nível de ação.

**Tabela 4:** Parâmetros laboratoriais urinários em adultos

Analitos urina 24 hs	Valor de referência	Nível de atenção	Nível de ação	Relevância
Volume	>2,0–2,5 L/d (25–30 mL/kg/d)	<1,5 L/d	<1 L/d	Adequação de ingestão líquida
Creatinina	Adultos < 50 anos 14–20 mg/kg/d (F) 20–25 mg/kg/d (M)	–	–	Adequação de coleta de 24hs
Cálcio	<150 mg/d (varia com dieta)	150–200 mg/d	>250 mg (F) >300 mg (M) >4 mg/kg/d (F/M)	Risco de litíase é contínuo com a calciúria
Ácido úrico	250–750 mg/d	–	>750 mg (F) >800 mg (M)	Avaliar junto com pH urinário
Oxalato	<45 mg/d (TFGe > 60 mL/min/1.73m^2^)	4–31 mg/d (F) 7–44 mg/d (M)	>40 mg (F) >45 mg (M)	>90 mg/d (1,0 mmol/d) sugere hiperoxalúria primária
Citrato	>320 mg/d	320–450 mg/d	<320 mg/d	Hipocitratúria ou acidose tubular renal distal
Sódio	50–100 mEq/d	100–150 mEq	150 mEq/d	Adequação da ingestão de sal
pH	*Urina 24hs* (não há referência)	Varia com dieta	<5,5 U24hs (Litíase úrica?) >7,0 U24hs (CaP, estruvita?)	–
	*Urina pós-jejum 12 hs* (2ª micção) Amostra Isolada (AI) pH < 5,8	–	pH > 5,8 (AI) e >6,2 U 24hs (excluir ATR)	Avaliar junto com HCO3 plasmático
Fósforo	400–1300 mg/d	Varia com dieta	–	FEP% + TmP (melhor em urina amostra isolada)
Potássio	20–100 mEq/d	Varia com dieta	–	TTKG (gradiente transtubular de potássio)
Ureia	15–40 g/d	–	–	Adequação da ingestão proteica
Magnésio	1.3–1.7 mg/kg/d	–	–	Baixa evidência
Cloro	110–250 mEq/dia	–	–	Ânion gap (urina)
Cistina	6.7–27.6 mg/d 28–115 μmol/d)	>0,8 mmoL/d	–	Cistinúria
Amônia	<50 mmol/d	–	–	Pouco disponível
Sulfato	20–80 mmol/d	–	–	Pouco disponível

Resumidamente, o volume urinário nos indica a adequação de ingestão líquida enquanto a excreção urinária de creatinina nos informa sobre a adequação da coleta. A determinação da calciúria em urina de 24 horas é importante no diagnóstico de hipercalciúria idiopática ou de causas monogênicas ou associada à ATR distal. Porém, o risco de litíase é contínuo com a calciúria, sem valores de corte^50^, assim como outros analitos^51^. Ainda não há consenso quanto à possível substituição pela dosagem de cálcio em urinas de amostra isolada para firmar esses diagnósticos em adultos^52^. A uricosúria só tem importância atual no contexto da litíase úrica se considerada em conjunto com o pH urinário em urina de 24 horas. A dosagem de oxalato urinário deve ser realizada em todo paciente com NL ou NC para auxiliar na diferenciação entre hiperoxalúria de causa secundária ou primária. Nesta última, caso o paciente apresente TFGe < 30 mL/min/1,73m^2^, deve-se proceder com a dosagem de oxalato plasmático conforme comentado anteriormente^43^. Recentemente, níveis elevados de oxalúria de 24 horas fora do contexto de HP1 têm sido relacionados à progressão para DRC^53^. A dosagem de citrato é importante para o diagnóstico de hipocitratúria idiopática ou associada aos quadros de acidose, como na ATR distal. O sódio urinário reflete a ingestão de sal. O pH em urina de 24 horas não tem valor de referência, variando com a dieta acidogênica ou não. Valores baixos de pH sugerem litíase úrica, relacionados à obesidade, e valores elevados estão rela­cionados a cálculos de fosfato de cálcio ou estruvita. Os valores de pH obtidos no sedimento urinário só têm valor quando reduzidos (menores do que 5,5), por afastarem ATR distal, pois valores mais altos, em faixa alcalina, podem ser oriundos de contaminação bacteriana. Para o diagnóstico de ATR distal, faz-se necessária uma coleta de urina de amostra isolada após jejum de 12 horas obtida na segunda micção, completado esse período de jejum. O exame deve ser obtido no mesmo dia da medida do HCO3 plasmático, acessando o estado de acidose, para o diagnóstico de ATR distal em sua forma completa. No caso de HCO3 normal, para complementar o diagnóstico de formas incompletas de ATR distal, indica-se a prova de sobrecarga com NH4Cl (cloreto de amônio). A dosagem do fósforo em urina de 24 horas reflete indiretamente a ingestão proteica e é utilizada em alguns índices de cristalização. Apesar de sua utilidade nas tubulopatias com fosfatúrias e raquitismos hipofosfatêmicos, o ideal nos estados fosfatúricos é o cálculo da fração de excreção de fósforo (FEP%), coletado juntamente com a creatinina em urina de amostra isolada e no sangue pela seguinte fórmula: FEP = [P (Urina) × Creatinina (Sérica)]/[P (Sérico) × Creatinina (Urinária)] × 100 e do cálculo da reabsorção tubular de fósforo [1 – FEP] × 100. Da mesma forma, a dosagem de potássio pode refletir a ingestão de potássio, mas, nos casos de hipocalemia, que pode ocorrer em tubulopatias associadas à nefrolitíase e/ou nefrocalcinose, o cálculo do TTKG (gradiente trans­tubular de K) diferencia as hipocalemias por perda renal (>4) das de origem gastrintestinal (<2) pela fórmula: [K (Urina) × Osm (Sérica)/K (Sérico) × Osm (Urina). A dosagem de ureia é útil para avaliação proteica calculada usando a fórmula do Equivalente Proteico do Aparecimento de Nitrogênio Urinário (PNA) através da fórmula PNA = (nitrogênio da ureia urinária + [0,031 × peso]) × 6,25, em que o nitrogênio da ureia urinária é igual a (ureia urinária/2,14 × volume urinário). Entretanto, ressalta-se que o PNA reflete a ingestão proteica total e que só a determinação do sulfato urinário indica a ingestão de proteína de origem animal. A dosagem de magnésio urinário tem poucas evidências de utilidade na avaliação do paciente com nefrolitíase, e sua implicação na terapêutica foi questionada no único estudo clínico empregando o hidróxido de magnésio. Sua importância refere-se à inclusão em fórmulas de cristalização urinária, como a de Tiselius^54^. O cloro tem pouca utilidade de maneira isolada, mas auxilia no cálculo do ânion gap urinário definido pelas concentrações urinárias de cloro subtraídas da soma Na+K, utilizadas como uma medida indireta de amônio (NH4) urinário^55^, este muito pouco disponível nos laboratórios, porém muito útil no diagnóstico de estados de acidose com ânion gap normal. A presença de cistina pode ser determinada qualitativamente pelo teste colorimétrico do nitroprussiato de sódio e comple­mentado pela determinação quantitativa de cistina, imprescindível para o diagnóstico da cistinúria. A dosagem do sulfato guarda boa correlação com a ingestão proteica, porém é indisponível em nosso meio, e a necessidade de sua determinação ainda permanece controversa.

Por fim, apesar da etiologia multifatorial, nos tempos atuais, com as novas técnicas de sequencia­mento, também devem ser buscadas causas monogên­icas de nefrolitíase, que podem estar presentes em até 30% das crianças e 10% dos adultos, atualmente com mais de 35 diferentes genes implicados^56^.

A Figura 3 sumariza as etapas de investigação e seguimento do paciente com nefrolitíase.

**Figura 3:** Investigação e seguimento do paciente com nefrolitíase.

### Resumo e Recomendações

Recomenda-se, de acordo com as diretrizes internacionais, que formadores de cálculos recorrentes, bilaterais ou múltiplos, formadores de cálculo único, porém de alto risco, pacientes com rim único, pacientes metabolicamente ativos ou com múltiplos fatores de risco sejam submetidos a uma avaliação metabólica completa e individualizada. Os principais fatores de risco considerados são início de nefrolitíase e/ou nefrocalcinose em idade precoce, histórico familiar, recorrência elevada, cálculos de brushita (fosfato de cálcio), ácido úrico ou de infecção (estruvita) (Grau de recomendação B; Nível de evidência 2).

A avaliação metabólica deve abranger coletas de sangue, urina de amostra isolada e urina de 24 horas. Os exames séricos devem ao menos contemplar dosagens de creatinina, Na, K, ácido úrico, HCO3, cálcio total + albumina ou cálcio ionizável e PTH. A urina de amostra isolada deve abranger o sedimento urinário e o pH de jejum. Em urina de 24 horas, os parâmetros mínimos a serem analisados envolvem a determinação do volume, creatinina, Ca, Na, K, oxalato, citrato, ácido úrico e pH. O teste qualitativo de cistina pode indicar a sua determinação quantitativa *a posteriori* (Grau de recomendação B; Nível de evidência 2).

Recomenda-se idealmente que ao menos duas amostras de urina de 24 horas sejam consideradas para o diagnóstico, sob dieta habitual, coletadas em 2 dias não consecutivos. O perfil metabólico basal deve ser obtido pelo menos 3 a 8 semanas após a última eliminação de cálculo, espontânea ou cirúrgica, sem infecção do trato urinário comprovada por urocultura (Graduação de recomendação B; Nível de evidência 2).

## Análise do Cálculo Urinário

O cálculo eliminado, sempre que possível, deve ser recuperado e submetido à análise. Conhecer a composição do cálculo urinário é importante para entender a fisiopatologia da doença, prevenir a recorrência e escolher a modalidade de tratamento clínico e/ou cirúrgico. Cálculos de brushita (fosfato dihidratado de cálcio), oxalato de cálcio monoidratado (COD) ou cistina são conhecidos por serem mais resistentes à fragmentação^40,44,57,58.59^. A coleta pode ser realizada diretamente pelo urologista, durante a retirada cirúrgica, ou pelo próprio paciente. Este deve ser instruído a filtrar a urina nos primeiros dias após uma crise álgica e, em caso de eliminação do cálculo ou de seus fragmentos, manter o material em recipiente seco e limpo até o encaminhamento para análise^60^.

### Análise Química

Embora seja a técnica mais utilizada em nosso meio, a análise química dos cálculos urinários apenas identifica a presença de íons e radicais individuais, sem ser capaz de quantificar ou diferenciar componentes específicos, como cálculos de purinas ou aqueles resultantes de medicamentos, por exemplo, ou mesmo associações de componentes, sendo atualmente considerada obsoleta^60^.

### Análise Cristalográfica

A espectroscopia infravermelha e a difração de raios-X são métodos qualitativos e quantitativos mais acurados para determinar a composição dos cálculos urinários e, portanto, considerados padrão-ouro. Os métodos físicos são normalmente precedidos de uma análise microscópica (estereomicroscópico) para caracterizar morfologicamente o cálculo, o que depende de treinamento especializado^61^. A espectroscopia infra­vermelha utiliza radiação infravermelha para causar vibrações atômicas e analisa as bandas de energia que surgem no espectro. Tem como inconvenientes a pouca disponibilidade em nosso meio, o alto custo e não constar ainda no rol dos exames realizados pelo Serviço Público de Saúde e Planos de Saúde. A difração de raios-X é uma técnica que utiliza raios monocro­máticos para identificar os constituintes do cálculo renal com base nos padrões de difração únicos produzidos pelo material cristalino, resultando em um difratograma característico. Embora seja uma técnica precisa, não é amplamente acessível para uso clínico regular devido à sua complexidade e ao custo elevado^60,62,63^.

### Recorrência

A análise do cálculo pode ser repetida em casos de litíase recorrente, apesar da terapia medicamentosa; quando houver recorrência precoce, após remoção completa do cálculo; e na recorrência tardia, após um longo período sem litogênese, porque a composição do cálculo pode mudar (mudanças na composição dos cálculos são citadas em até 21% dos casos)^64^.

### Resumo e Recomendações

Cálculos coletados pelos pacientes ou retirados no momento da intervenção cirúrgica devem ser encami­nhados para análise morfológica associada a um método físico ou cristalográfico, idealmente por meio da espectroscopia infravermelha ou difração de raios-X (Grau de recomendação C; Nível de evidência: 3).

Sugere-se repetição da análise do cálculo em pacientes com cálculos recorrentes apesar da terapia medicamentosa; em recorrência precoce após remoção completa do cálculo e em recorrência tardia após um longo período sem cálculo (Grau de recomendação C; Nível de evidência 3).

## Investigação e Monitorização Radiológica

### Exames de Imagem do Trato Urinário

A tomografia computadorizada (TC) sem uso de contraste é a técnica de imagem mais precisa para identificar cálculos urinários graças às suas elevadas sensibilidade e especificidade, bem como à capacidade de medir com precisão o tamanho dos cálculos e avaliar outras patologias não relacionadas aos cálculos^65^.

A TC de baixa dose possui muitas das mesmas vantagens da TC padrão e reduz a exposição à radiação; no entanto, sua precisão diagnóstica é reduzida em pacientes obesos e a modulação da dose deve ser considerada^66^.

A TC também pode fornecer informações relativas à composição dos cálculos. Há uma medida de atenu­ação, as unidades Hounsfield (UH), que descreve a densidade dos tecidos e substâncias. Em uma escala, a água tem a densidade de 0 (zero) UH e o osso denso de 1000 UH. Cálculos de ácido úrico tem densidade entre 200 e 400 UH, enquanto cálculos de oxalato de cálcio têm densidade entre 600 e 1200 UH (2). Mais recentemente, a utilização da TC de dupla energia tem-se mostrado superior à TC convencional na diferenciação entre cálculos de ácido úrico e cálculos compostos por cálcio, com alguns estudos relatando sensibilidades e especificidades próximas a > 95%^67^.

Em adultos, comparativamente à tomografia com­putadorizada sem contraste (exame de referência, com sensibilidade > 95%), a sensibilidade da ultrassono­grafia foi de 60% e a especificidade, de 90%. Contudo, quando essas modalidades de imagem foram submetidas a um ensaio clínico randomizado, constatou-se que apresentavam acurácia diagnóstica equi­valente no contexto do departamento de emergência^17^.

A radiografia simples do rim, ureter e bexiga (RUB) é mais útil na avaliação do crescimento intervalar de cálculos em pacientes com doença calculosa conhecida e tem menor utilidade na detecção de cálculos na crise aguda, em atendimento de emergência (sensibilidade de 58% e especificidade de 90%), quando comparados à tomografia^66^.

A sensibilidade da ressonância magnética é variável, em torno de 82%. E assim como na ultrassonografia, pode ser maior pela presença de hidronefrose associada. Quando os cálculos são visualizados na ressonância magnética, a especi­ficidade é alta (98,3%). Em geral, a ressonância magnética custa mais do que uma CT, além de ter tempos de aquisição de imagens muito mais longos^66^.

Na gestação, a ultrassonografia pode ser utilizada como ferramenta diagnóstica, quando necessário. No entanto, alterações fisiológicas da gestação podem mimetizar obstrução ureteral. A ressonância magnética pode ser utilizada como segunda opção, a fim de definir o nível da obstrução e visualizar cálculos (aparecem como defeito de enchimento)^68^.

A recorrência sintomática de um cálculo pode variar desde um desconforto mínimo com a passagem espontânea do cálculo até uma dor intensa^69^. A recorrência radiográfica, incluindo a formação de novos cálculos e o crescimento dos cálculos exist­entes, pode ser um substituto útil e prático para a recorrência sintomática. Infelizmente, nem a resolução dos sintomas nem os relatos do paciente sobre a passagem bem-sucedida de cálculos urinários são sempre confirmatórios^70^.

Não há estudos que avaliem a frequência com que os diagnósticos de imagem devam ser repetidos em pacientes que têm ou tiveram cálculos renais ou ureterais. Sugere-se, a partir do consenso entre especialistas, a estratificação dos pacientes^69^.

Em uma revisão sistemática e metanálise realizada pela Associação Europeia de Urologia com 5467 pacientes submetidos a intervenção e “livres de cálculos”, estimou-se que, para uma margem de segurança de 80%, os pacientes deveriam fazer exames de imagem de seguimento por pelo menos 2 anos (para pacientes com cálculos radiopacos) e por pelo menos 3 anos (para pacientes com cálculos radiotransparentes). Para uma margem de 90%, o seguimento deveria ser feito por 5 anos, em pacientes nos quais não houve recorrência de cálculos^71^.

Pacientes com “fragmentos residuais” com fragmentos < 4 mm deveriam fazer exames de imagem de seguimento por 4 anos, uma vez que, num período de 29 meses, as taxas de necessidade de reintervenção urológica nesse grupo variaram entre 17% a 29%, progressão de doença entre 9% a 34% e passagem espontânea de cálculo entre 21% a 34%. Nos pacientes com fragmentos > 4 mm, deve-se oferecer nova intervenção urológica definitiva, uma vez que a taxa de sucesso é alta (24% a 100%)^71^.

Não há dados suficientes para “pacientes com anormalidades metabólicas – alto risco”. Conforme orientação estabelecida em consenso, após extensa discussão de painel de especialistas da Sociedade Europeia de Urologia, em pacientes com anormali­dades metabólicas sem tratamento medicamentoso dirigido, menos de 40% permaneceram sem cálculos num seguimento de 3 anos. Por isso, esse tempo de seguimento é proposto para esse grupo de pacientes^71^.

A Figura 4 resume o tempo de seguimento sugerido para essas diversas situações clínicas.

**Figura 4:** Recomendação de seguimento radiológico de pacientes com diagnóstico de litíase urinária assintomáticos.

Notes – *Livre de cálculos: sem fragmentos em exame de imagem pós-operatório; **Alto risco: anormalidade bioquímica identificada (hipercalciuria, hipocitraturia, hiperuricosuria, acidose tubular renal) ou cálculos de alto risco, como estruvita ou cistina.

### Resumo e Recomendações

A resolução dos sintomas, bem como os relatos do paciente sobre a eliminação de cálculos urinários, nem sempre é confirmatória (Grau de recomendação: D; Nível de evidência: 4).

Recomenda-se acompanhamento por meio de imagens para confirmar a passagem do cálculo (Grau de recomendação: B; Nível de evidência: 2).

Os exames de imagem propostos para seguimento incluem raios-X simples de abdome (Rx) e ultrassono­grafia, baseando-se nas características dos cálculos e preferências do médico assistente (Grau de recomen­dação: B; Nível de evidência: 2).

A tomografia computadorizada deve ser reservada para pacientes sintomáticos ou em avaliação pré-operatória, para minimizar exposição à radiação (Grau de recomendação: C; Nível de evidência: 3).

### Investigação de Distúrbio Mineral Ósseo em Pacientes com Litíase Renal

A nefrolitíase está associada a um aumento no risco de redução de massa óssea. Vários estudos relataram densidade mineral óssea (DMO) diminuída em portadores de nefrolitíase quando comparados a indivíduos saudáveis e maior risco de fraturas^72,73,74,75,76,77^. A desmineralização óssea pode estar relacionada, entre outros fatores, a menor ingestão de cálcio e a maiores níveis de cálcio na urina, mas foi evidenciada também em normocalciúricos ou litiásicos não categorizados de acordo com a excreção urinária de cálcio^78,79,80,81,82,83^.

Uma metanálise de 28 estudos caso-controle comparou 1595 pacientes litiásicos (LIT) a 3402 indivíduos sadios controles (CONT) na quarta década de vida analisando a diferença padronizada das médias (*standardized mean difference*, SMD). Demonstrou-se que os LIT apresentaram valores mais baixos de escore T na coluna lombar (em 7 estudos, SMD = -0,69), no fêmur total (em 7 estudos, SMD = -0,82) e no colo do fêmur (em 6 estudos, SMD = -0,67). Em relação ao risco de fraturas, a análise dos dados de 4 estudos de caso-controle demonstrou um aumento significativo no risco de fraturas entre os LIT (Razão de chance, *Odds ratio*, OR = 1,15). Essa tendência também foi observada em 2 estudos longitudinais (OR = 1,31). Além disso, os LIT apresentaram um risco 4 vezes maior de desenvolver osteoporose em comparação com os CONT (OR = 4,12)^84^.

Na avaliação metabólica de pacientes litiásicos, sugere-se a investigação de condições ou doenças subjacentes que se associam mais frequentemente a distúrbios minerais ósseos, tais como hipercalciúria idiopática, acidose tubular renal distal (completa ou incompleta), nefrocalcinose e/ou rim espongiomedular, hiperparatireoidismo primário, síndromes de má absorção ou indivíduos submetidos à cirurgia bariátrica, tubulopatias ou doenças monogênicas^56^.

Recomendações dietéticas (adequação da ingestão proteica, de sódio e de cálcio), reposição de vitamina D e terapias farmacológicas indicadas para redução da formação de cálculos (tiazídicos, citrato ou álcalis) podem contribuir para estabilizar a perda óssea^36^.

Nota: O padrão de referência para diagnóstico de osteoporose pela Organização Mundial da Saúde (WHO) corresponde a um T-score ≤ 2,5 em colo de fêmur (o escore T foi calculado em mulheres brancas 20–29 anos, banco de dados do NHANES). Osteoporose pode ser diagnosticada em mulheres na pós-menopausa e em homens com 50 ou mais anos de idade se o T-score for ≤ 2,5 em coluna lombar, colo de fêmur ou fêmur total. De acordo com os novos critérios diagnósticos da Organização Mundial da Saúde (WHO), o termo “osteopenia” não deve mais ser utilizado, devendo ser substituído por “baixa massa óssea” ou “baixa DMO”, já que esse achado não necessariamente indica maior risco de fratura. Em mulheres pré-menopausa, crianças e homens com menos de 50 anos de idade deve ser utilizado o escore Z (e não o escore T): Z-score ≤ 2,0 é definido como baixa massa óssea para idade cronológica^85^.

### Resumo e Recomendações

Indivíduos com nefrolitíase apresentam maior risco de doença metabólica óssea e de fraturas (Grau de recomendação B; Nível de evidência 2). Na avaliação metabólica de pacientes litiásicos sugere-se investigar condições ou doenças subjacentes que se associam mais frequentemente a distúrbios minerais-ósseos (Grau de recomendação C; Nível de evidência 3).

Pacientes litiásicos, especialmente os com hiper­calciúria, devem ser avaliados para risco de osteoporose ou perda de massa óssea por meio de densitometria óssea, em aparelho de absorciometria de raios X de dupla energia (DEXA)* (Grau de recomendação B; Nível de evidência 2).

## Tratamento

### Recomendações Nutricionais

A dieta desempenha um papel central na gênese e prevenção de cálculos renais. Embora o tratamento farmacológico possa ser necessário para alguns pacientes, a intervenção nutricional pode ser suficiente para diminuir o risco de litíase urinária em muitos pacientes. Não existe uma dieta especializada única para a prevenção de cálculos, mas várias recomendações e princípios são respaldados por estudos de intervenção e epidemiológicos ou observacionais.

A Tabela 5 resume as orientações nutricionais gerais para pacientes litiásicos, assim como aquelas relacionadas a distúrbios metabólicos específicos associados à nefrolitíase.

**Tabela 5:** Recomendação nutricional de acordo com os fatores de risco e distúrbios metabólicos

Fatores de risco/distúrbios metabólicos	Recomendação nutricional	Nível de recomendação	Referências
Obesidade	Adequação do Índice de Massa Corpórea (IMC)	A	86,87,88
	Redução calórica balanceada e adequada à necessidade de perda de peso/paciente	D	
	Ingestão de proteína: 0,8 a 1,0 g/kg de peso corporal ideal/dia (evitar dietas hiperproteicas (*low-carb*) com potencial ácidogênico)			
Síndrome Metabólica (SM)/diabetes *mellitus* tipo 2/obesidade/hipertensão/dislipidemia/hiperuricemia	Recomendação dietética com foco no critério diagnóstico de SM do paciente	D	89 90,91
	Redução calórica com adequação de proteína animal/redução de alimentos fonte em colesterol/gordura saturada e de alto índice glicêmico/↓ ingestão de sal (NaCl) <6 g/dia ↑↑ consumo de vegetais e frutas (dieta alcalina)		
Cirurgia Bariátrica	Ingestão de cálcio: 1000 a 1200 mg/dia (considerar suplementação*)	D	92
	↓ Ingestão de alimentos ricos em oxalato; ↓ ingestão de sal (NaCl) < 6 g/dia		
	↓ Ingestão de gordura: 25–30% do valor energético total da dieta		
	↑> Ingestão de líquidos (30 mL/kg/dia + 400 mL perdas insensíveis): diurese 2,5 a 3,0 L/dia		
	Ingestão de proteína: 0,8 a 1,0 g/kg de peso corporal ideal/dia		
	↑ Consumo de vegetais e frutas		
Baixo volume urinário	↑ Ingestão de líquidos (30 mL/kg/dia + 400 mL perdas insensíveis): diurese 2,5 a 3,0 L/dia	A	93,94,95,96
Hipercalciúria	↓ Ingestão de sal (NaCl) < 6 g/dia	A	96,97,98,99,100
	Ingestão de proteína: 0,8 a 1,0 g/kg de peso corporal ideal/dia		
	↑ Consumo de vegetais e frutas/Adequação do IMC		
	Ingestão de cálcio: 1000 a 1200 mg/dia*	D	
Hiperoxalúria secundária	Ingestão de cálcio: 1000 a 1200 mg/dia*	A	97,101,102,103,104
	↓ Ingestão de alimentos ricos em oxalato	B	
	Ingestão de proteína: 0,8 a 1,0 g/kg de peso corporal ideal/dia	D	
pH urinário ácido/Hiperuricosúria	↑ Ingestão de líquidos (30 mL/kg/dia + 400 mL perdas insensíveis): diurese 2,5 a 3,0 L/dia	D	97,105,106,107,108
	Ingestão de proteína: 0,8 a 1,0 g/kg de peso corporal ideal/dia com ↓ proteína fonte animal ↑ consumo de vegetais e frutas (dieta + alcalina) – padrão de dieta DASH		
	Adequação do IMC (vide recomendação para obesidade acima)		
	*Obs.: s/ evidências benéficas* ↓ *purinas em litíase úrica /* ↓ *consumo de álcool e especialmente cerveja para gota* ^109^		
Hipocitratúria	Ingestão de proteína: 0,8 a 1,0 g/kg de peso corporal ideal/dia com ↓ proteína fonte animal ↑ consumo de vegetais e frutas (dieta + alcalina) – padrão de dieta DASH	A	98,105, 110,111,112,113
	↑ Consumo de alimentos ricos em citrato/malato (ex.: limão, laranja, tangerina, melão)	B	
Cistinúria	↑ Ingestão de líquidos p/ diurese ≥ 3,0 L/24 h e ↓ concentração de cistina < 250 mg/L consumo de vegetais e frutas (dieta + alcalina) com padrão de dieta DASH	D	114,115,116
	Ingestão de proteína 0,8 a 1,0 g/kg de peso corporal ideal/dia/Ingestão de sal: < 6 g/dia		
Litíase em geral *ou* litíase sem distúrbio metabólico identificado	↑ Ingestão de líquidos (30 mL/kg/dia + 400 mL perdas insensíveis): diurese 2,5 a 3,0 L/dia	A	86 87,88,94, 105,117,118,119,120,121,122,123,124,,125
	Adesão a um padrão de dieta DASH ou Mediterrânea		
	Ingestão de cálcio: 1000 a 1200 mg/dia		
	Ingestão de proteína: 0,8 a 1,0 g/kg de peso corporal ideal/dia		
	↓ Ingestão de sal (NaCl): < 6 g/dia		
	↑ Consumo de vegetais e frutas		
	Adequação do IMC/↓ alimentos ricos em oxalato (controverso)	A/D	

### Suplementos Alimentares, Vitaminas e Probióticos

Os suplementos de cálcio, diferentemente do cálcio proveniente da alimentação, podem estar associados a um aumento de até 20% no risco de desenvolvimento de cálculos renais. Isso ocorre em virtude de sua ingestão distante das refeições, que não exerce um efeito quelante significativo sobre o oxalato, ou ainda devido ao acúmulo da suplementação de cálcio somado à ingestão dietética de cálcio^121^. Recomenda-se, portanto, dar prioridade à melhoria da alimentação mediante o aumento do consumo de alimentos ricos em cálcio (Grau de Recomendação D). O consumo de suplementos de vitamina C em doses superiores a 1000 mg por dia foi relacionado a um acréscimo de 16% na ocorrência de cálculos renais, bem como a aumentos nos níveis de oxalato urinário em homens^126^. Estudos de intervenção com doses de 1000 e 2000 mg por dia também revelaram aumentos de até 61% nos níveis de oxalúria^127,128^. Dessa forma, é aconselhável evitar o uso de suplementos (Grau de Recomendação **D**). A suplementação de vitamina D pode aumentar a calciúria e o risco de nefrolitíase^129, 130^. Porém, os estudos nessa área apresentam resultados divergentes^131,132,133^. Consequentemente, é fundamental avaliar de forma personalizada a suplementação de vitamina D em pacientes litiásicos, considerando a necessidade individual, os níveis de cálcio sérico e o efeito no cálcio urinário (Grau de Recomendação **D**). Indivíduos litiásicos parecem apresentar diversidade microbiana intestinal reduzida, incluindo menor colonização pelo *Oxalobacter formigenes*, que desempenha um papel crucial na degradação do oxalato dietético^134^. Apesar da possível ligação com a redução dos níveis de oxalato na urina, a suplementação de *O. formigenes* não demonstrou de maneira conclusiva esse efeito benéfico^135,136^. Além do *Oxalobacter formigenes*, cepas de *Lactobacillus* e *Bifidobacterium* também têm a capacidade de metabolizar o oxalato presente na dieta, o que limitaria sua absorção e subsequente excreção urinária^137^. No entanto, os resultados de estudos clínicos randomizados têm sido contraditórios^138,139,140,141^. A prescrição individualizada pode ser considerada como uma abordagem alternativa e adjuvante, que requer investigações adicionais em relação a dosagem, tipo e duração da administração (Grau de Recomendação **D**). Os suplementos de proteína são amplamente utilizados para aumentar a massa muscular e aprimorar o desempenho durante o exercício físico. O consumo de *whey protein* (proteína do soro do leite) ou de albumina por indivíduos saudáveis, em um curto período e sob dieta controlada, não demonstrou alterações significativas nos fatores relacionados à litogênese^142^. Contudo, à luz da recomendação atual de adotar dieta com níveis normais de proteína para pacientes litiásicos, é crucial avaliar de maneira individualizada a prescrição de suplementos proteicos, levando em conta as necessidades específicas e a monitorização bioquímica urinária (Grau de Recomendação **D**).

### Resumo e Recomendações

As recomendações dietéticas, de maneira geral, incluem a ingestão adequada de líquidos, consumo moderado de cálcio, baixo teor de sódio e restrição de proteínas de origem animal na dieta (Grau de recomendação B; Nível de evidência: 2).

As recomendações podem ser direcionadas ao perfil metabólico identificado, mas adequação do peso e adesão a um padrão de dieta DASH ou Mediterrânea são úteis, independentemente destes (Grau de recomendação B; Nível de evidência: 2).

Os efeitos da prescrição de suplementos e vitaminas permanecem controversos, devendo ser testados e analisados individualmente (Grau de recomendação D; Nível de evidência: 4).

### Tratamento Farmacológico

#### Tiazídicos

Os diuréticos tiazídicos devem ser oferecidos a pacientes com alta concentração urinária de cálcio e/ou recorrência de litíase urinária cálcica. Embora o mecanismo preciso de ação dos tiazídicos ou tiazídicos-like (clortalidona; indapamida) não tenha sido elucidado completamente, eles seriam apropriados quando medidas dietéticas e aumento da ingestão de líquidos não são suficientes para controlar a recorrência de litíase renal e nos casos de perda de massa óssea^143^. Nessas circunstâncias, essas drogas são consideradas apropriadas tanto para cálculos de oxalato de cálcio quanto de fosfato de cálcio.

Embora os efeitos dos diferentes tiazídicos sejam relativamente semelhantes em termos de prevenção da recorrência do cálculo, sua potência e seus efeitos colaterais podem ser diferentes^144^. No entanto, faltam estudos clínicos mais completos que incluam pacientes hipercalciúricos com o objetivo de comparar potência e efeitos colaterais, empregando diferentes doses e com seguimento de longo prazo^145^. As doses e os regimes sugeridos incluem hidroclorotiazida 25 mg duas vezes ao dia ou 50 mg ao dia, clortalidona 25–50 mg uma vez ao dia ou indapamida SR 1,5 mg uma vez ao dia^144^.

Recomenda-se fortemente a monitorização dos níveis séricos de potássio ao usar essas drogas para evitar o aumento do risco cardiovascular e minimizar a intolerância à glicose, dentre outros efeitos colaterais. Geralmente, a associação de drogas, como citrato de potássio ou amilorida, pode minimizar a hipocalemia. Em termos gerais, a hidroclorotiazida tem a menor meia-vida, em torno de 8–12 horas, o que demandaria administração de 12/12 horas para manter controle de calciúria ao longo das 24 horas. Porém, usar diurético à noite não seria tão adequado, pelo risco de impacto na qualidade de sono e risco de queda, sobretudo em idosos. Sob esse ponto de vista, a clortalidona ou indapamida (infelizmente não disponíveis no SUS) seriam mais indicadas, pois têm meia-vida mais longa, em torno de 24–36 horas.

Um efeito colateral potencial, altamente debatido, observado após o uso em longo prazo de tiazídicos ou tiazídicos-like, é sua associação com maior risco de câncer de pele, especialmente carcinoma cutâneo e labial de células escamosas, devido à sua propriedade fotossensibilizante^143,146^.

Vários ensaios clínicos já demonstraram, ainda que com número pequeno de pacientes, que os tiazídicos são efetivos na prevenção de recorrência de cálculo renal. Entretanto, o papel dessa classe de medicamentos foi recentemente questionado com a publicação do estudo suíço NOSTONE, que randomizou 416 pacientes com nefrolitíase cálcica recorrente para hidroclorotiazida (em doses diárias de 12,5 mg, 25 mg ou 50 mg) ou placebo^147^. O desfecho primário foi um composto de recorrência sintomática ou radiológica de cálculos renais, com duração mediana de acompanhamento de 2,9 anos. Os resultados não demonstraram diferenças estatisticamente significativas no desfecho primário entre hidroclorotiazida em qualquer dose e placebo, gerando controvérsia significativa sobre a permanência das tiazidas no arsenal de prevenção de cálculos. Entretanto, vários pontos podem ter enviesado o NOSTONE para um resultado nulo, incluindo o uso de uma tiazida de ação mais curta administrada uma vez ao dia, variações dietéticas que potencialmente afetam o efeito do medicamento, não terem sido incluídos apenas pacientes com hipercalciúria, a duração relativamente curta do acompanhamento e o desfecho primário considerando a passagem de cálculos existentes como um novo evento de cálculo^148^. Em uma análise *post hoc*, ao considerar exclusivamente a formação ou o crescimento de novos cálculos como desfecho primário, os dois grupos que receberam as doses mais altas de hidroclorotiazida demonstraram reduções significativas no risco de formação de cálculos^149^.

#### Bicarbonato de sódio e citrato de potássio

O bicarbonato, os sais de citrato e outros metabólitos do ciclo do ácido cítrico geram uma carga alcalina no organismo. A ação do bicarbonato é imediata, pois ele neutraliza o ácido gástrico e, quando presente em excesso em relação à secreção ácida, é absorvido^150^. O citrato é metabolizado como ácido cítrico com captação de um próton, levando à formação de novo bicarbonato no sangue. As células do túbulo proximal renal podem excretar o álcali extra como bicarbonato, aumentando o pH da urina, ou como citrato, que não altera o pH urinário. Este último ocorre porque a reabsorção do citrato filtrado é reduzida pela carga alcalina^151^. A carga alcalina também reduz o cálcio na urina, aumentando a reabsorção de cálcio nos túbulos renais. No entanto, isso é verdadeiro apenas para os sais de potássio; o álcali de sódio pode não ser tão eficaz.

Tanto os sais alcalinos de sódio quanto de potássio podem aumentar efetivamente os níveis de citrato urinário; contudo, o citrato de potássio é geralmente preferido ao citrato de sódio. Essa preferência deve-se ao potencial do sódio em aumentar os níveis de cálcio urinário, o que pode levar ao aumento da calciúria^152^.

O citrato de potássio é uma medicação adjuvante no tratamento da hipercalciúria, pois reduz a saturação urinária ao se complexar com o cálcio tanto no intestino quanto possivelmente nos rins^153^. Além disso, o citrato aumenta a dissociação do ácido úrico, diminuindo a chance de sua precipitação e impedindo a formação de cálculos mistos com o oxalato de cálcio. Evidências na literatura sugerem ainda que agentes alcalinizantes inibem a reabsorção óssea, melhoram a massa óssea, induzem hipocalciúria e estimulam a atividade osteoblástica^151^. A dose terapêutica diária varia de 20 a 60 mEq, de acordo com a tolerância de cada paciente. Em casos especiais, como pacientes com cistinúria, a dose pode ser aumentada até 90 mEq/dia.

No mercado brasileiro, o citrato de potássio é o mais disponível e indicado. Pode ser encontrado em formulações prontas ou em farmácias de manipulação. Geralmente está disponível em comprimidos ou cápsulas de liberação lenta de 5, 10 e 15 mEq.

Um grande limitante do uso dessa medicação é a intolerância gástrica e a doença do refluxo gastroesofágico, que pode ser minimizada com a ingestão após as refeições^154^. Atenção especial deve ser dada a pacientes com doença renal crônica devido ao risco de hipercalemia ou alcalose metabólica, especialmente se associada ao uso de outros álcalis, como bicarbonato de sódio.

#### Alopurinol

O alopurinol é uma medicação utilizada na prevenção da nefrolitíase em pacientes com cálculos oxalato de cálcio ou de ácido úrico, especialmente quando associados à hiperuricosúria (excreção urinária de ácido úrico > 800 mg/dia)^155^. Entretanto, nesta última situação, deve-se concomitantemente manter o pH urinário alcalino, para obter maior solubilidade do ácido úrico.

O alopurinol atua como inibidor da xantina oxidase, uma enzima que converte hipoxantina em xantina e, subsequentemente, xantina em ácido úrico. Ao inibir essa enzima, o alopurinol reduz a produção de ácido úrico, resultando em menores concentrações de ácido úrico no sangue e na urina^156^. Os benefícios na prevenção de cálculos renais incluem redução da saturação de ácido úrico, o que diminui a formação de cristais de ácido úrico que podem servir como núcleo para a formação de cálculos. Além disso, o ácido úrico pode formar cristais que servem de núcleo para a precipitação de oxalato de cálcio. Ao reduzir a concen­tração de ácido úrico, o alopurinol também pode ajudar a prevenir a formação de cálculos de oxalato de cálcio^157^.

Ettinger et al., em 1986, randomizaram 60 pacientes com nefrolitíase recorrente, hiperuricosúria e normocalciúria, para receber alopurinol (100 mg três vezes ao dia) ou placebo. Após o estudo, o grupo placebo apresentou 63,4% menos cálculos (P < 0,001), enquanto o grupo alopurinol teve 81,2% menos cálculos (P < 0,001). Durante o período do estudo, a taxa média de eventos de cálculos foi de 0,26 por paciente por ano no grupo placebo e 0,12 no grupo alopurinol^158^.

A dosagem de alopurinol é geralmente individu­alizada com base nos níveis de ácido úrico sérico e urinário, bem como na função renal do paciente. A dose típica varia de 100 a 300 mg por dia, podendo ser ajustada, conforme necessário. Os efeitos adversos podem incluir erupções cutâneas, efeitos gastroin­testinais e, raramente, reações de hipersensibilidade graves, como a síndrome de Stevens-Johnson^159^.

#### Gliflozinas

As gliflozinas, também chamadas de inibidores do cotransportador de sódio-glicose (iSGLT2), são medicamentos que agem nos rins, impedindo a reabsorção de glicose nos túbulos renais, promovendo eliminação de glicose pela urina. Esses fármacos demonstraram em vários estudos redução significativa dos desfechos cardiovasculares e renais em pacientes diabéticos e não diabéticos^160^.

Em estudos não especificamente desenhados para avaliar o efeito das gliflozinas, notou-se aumento do volume urinário, maior citratúria, diminuição do pH urinário, efeitos anti-inflamatórios e antifibróticos, com supressão da expressão de marcadores de macrófagos (estudos *in vivo* e *in vitro*), diminuição do ácido úrico sérico, com aumento da uricosúria e diminuição da supersaturação urinária para o fosfato de cálcio em indivíduos saudáveis^161,162^.

Embora haja resultados preliminares encoraja­dores com os iSGLT2, esses medicamentos precisam ser testados em pacientes com litíase renal, com foco principal na redução dos cálculos urinários. Uma abordagem viável neste momento seria considerar a associação de gliflozinas em pacientes com litíase renal que já tenham indicações estabelecidas para esses medicamentos, como na presença de doença renal diabética, por exemplo^163^.

#### D-penicilamina e tiopronina

A cistinúria é uma doença hereditária recessiva, caracterizada por um defeito na reabsorção de aminoácidos dibásicos (cistina, ornitina, lisina e arginina), nas células dos túbulos renais e intestinais. A cistina é um homodímero formado pela ligação de duas moléculas de cisteína através de uma ligação dissulfeto. Medicamentos contendo tiol (por exemplo, tiopronina [2-mercaptopropionilglicina], D-penicilamina) possuem grupos sulfidrila que podem quebrar essa ligação dissulfeto, resultando na formação de dissulfetos mistos de fármaco-cisteína, os quais são mais solúveis do que o homodímero de cistina^115^. Para pacientes com cistinúria que não respondem bem às medidas conservadoras, como aumento importante da diurese, alcalinização vigorosa com citrato de potássio e redução na ingestão de sódio e metionina na dieta^115^, ou que possivelmente serão resistentes a elas, recomenda-se o tratamento específico com tiopronina, pela menor incidência de efeitos adversos do que a D-penicilamina^114,116^, mas o inconveniente é a indisponibilidade da primeira no Brasil. A dose de tiopronina para adultos varia de 600 a 900 mg/dia (dividida em 3 tomadas). A dose habitual de Cuprimine® (D-penicilamina em cápsulas de 250 mg) para adultos varia de 500 a 1.500 mg/dia, distribuída em 2 a 3 doses. A dose diária deve ser ajustada para manter a concentração de cistina não ligada na urina abaixo de 250 mg/L (1 mmol/L). Se a D-penicilamina for utilizada em longo prazo, é importante suplementar com piridoxina (vitamina B6) a uma dose de 50 mg por dia, pois a D-penicilamina pode causar deficiência dessa vitamina.

Os medicamentos contendo tiol podem causar uma variedade de efeitos adversos, como febre, erupção cutânea, alteração do paladar, artrite, leucopenia, anemia aplástica, hepatotoxicidade e deficiência de piridoxina (vitamina B6). Além disso, os pacientes podem apresentar proteinúria, geralmente associada à nefropatia membranosa, raramente glomerulonefrite crescente ou doença de lesão mínima^116^.

#### Piridoxina (vitamina B6) e lumasirana

A piridoxina atua como cofator para a enzima alanina-glioxilato aminotransferase (AGT). Essa enzima é responsável pela conversão de glioxilato em glicina no fígado. Portanto, ao facilitar a conversão de glioxilato em glicina, a piridoxina ajuda a diminuir a quantidade de oxalato produzido no corpo. Aproximadamente até 50% dos pacientes com hiperoxalúria primária do tipo 1 (PH1) responderão à terapia com piridoxina com uma redução significativa da excreção urinária de oxalato, e isso ocorre mais comumente em pacientes com mutações homozigóticas no AGXT p.Gly170Arg ou p.Phe152Ile^164^. A dose da piridoxina é de 5–20 mg/kg dividida em uma ou duas doses por dia. A resposta considerada positiva é definida como uma redução no oxalato urinário superior a 30%^43^.

A lumasirana faz parte de uma nova classe de medicamentos utilizada para tratar a PH1, representada por um RNA de interferência (RNAi) cujo alvo é a degradação do RNA mensageiro do gene HAO1 (hidroxiacido-oxidase 1), que codifica a enzima glicolato oxidase (GO), que converte glicolato em glioxilato e o glioxilato em oxalato. Ao inibir a HAO1, a lumasirana reduz a produção de GO, o precursor direto do oxalato no fígado. É administrado por via subcutânea e a dosagem calculada com base no peso corpóreo. Um consenso recente da OxalEurope, em conjunto com a Rede Europeia de Doenças Raras (ERKNet) e várias outras entidades, apresenta as opções e adequação dessas novas terapias, bem como da terapia convencional em HP1. A droga já foi aprovada pelo FDA, Agência Europeia de Medicamentos e pela ANVISA com base nos estudos pivotais de fase 3 em crianças e adultos incluindo até pacientes com estágios mais avançados de doença renal crônica ou até mesmo em diálise^43^. Entretanto, a droga ainda não está disponibilizada para uso em nosso meio e o custo elevado da medicação ainda compromete o acesso de tratamento à população, como é o caso de outras drogas órfãs.

#### Outras medicações

No caso de cálculos coraliformes, a terapia antibiótica, associada à remoção cirúrgica, é o componente primário para o tratamento dos cálculos de estruvita. A esterilização da urina com terapia antibiótica pode ajudar a diminuir a taxa de recorrência de cálculos. No passado, o ácido acetohidroxâmico era indicado para tratar pacientes com fragmentos residuais ou cálculos recorrentes de estruvita pós-cirurgia ou aqueles que não podiam ser submetidos à cirurgia^165^. No entanto, esse agente está associado a efeitos colaterais graves que limitam a sua utilização, que já é muito rara em nosso meio.

A Figura 5 ilustra o tratamento de acordo com o tipo de cálculo (por análise, imagem ou presumido) e/ou distúrbio metabólico.

**Figura 5:** Tratamento de acordo com o tipo de cálculo ou distúrbio metabólico.

Notas – *Investigar causa; **Teste genético.

### Resumo e Recomendações

Diuréticos tiazídicos podem ser administrados a pacientes com hipercalciúria e nefrolitíase cálcica recorrente (Grau de recomendação: B; Nível de evidência: 2).

Citrato de potássio é recomendado para pacientes com hipocitratúria e nefrolitíase cálcica recorrente (Grau de recomendação: A; Nível de evidência: 1).

Diuréticos tiazídicos e/ou citrato de potássio podem ser administrados a pacientes com cálculos recorrentes de cálcio sem alterações metabólicas (Grau de recomendação: B; Nível de evidência: 2).

Deve-se oferecer citrato de potássio a pacientes com cálculos de ácido úrico e cistina para elevar o pH urinário a um nível ótimo (acima de 6,5) (Grau de recomendação: B; Nível de evidência: 2).

Em pacientes com cálculo de ácido úrico, o alopurinol pode ser usado como terapia adjuvante em pacientes com hiperuricemia ou hiperuricosúria (Grau de recomendação: B; Nível de evidência: 2).

Medicamentos contendo tiol podem ser oferecidos a pacientes com cálculos de cistina que não respondem às modificações dietéticas e à alcalinização urinária (Grau de recomendação: C; Nível de evidência: 3).

Na hiperoxalúria entérica, suplementos de cálcio com as refeições e citrato alcalino podem ser prescritos (Grau de recomendação: B; Nível de evidência: 2).

É recomendado testar a resposta à piridoxina em portadores de hiperoxalúria primária tipo 1. Nesses pacientes, a lumasirana pode ser indicada, de preferência com auxílio de um especialista (Grau de recomendação: B; Nível de evidência: 2).
